# Effects of Anti-Diabetic Drugs on Fracture Risk: A Systematic Review and Network Meta-Analysis

**DOI:** 10.3389/fendo.2021.735824

**Published:** 2021-10-14

**Authors:** Yu-Sheng Zhang, Yan-Dan Zheng, Yan Yuan, Shi-Chun Chen, Bao-Cheng Xie

**Affiliations:** ^1^ Department of Pharmacy, The First People’s Hospital of Foshan, Foshan, China; ^2^ Department of Clinical Laboratory, The First People’s Hospital of Foshan, Foshan, China; ^3^ Affiliated Dongguan Hospital, Southern Medical University, Dongguan, Guangdong, China

**Keywords:** anti-diabetic drug, fracture, type 2 diabetes mellitus, systematic review, meta-analysis

## Abstract

**Purpose:**

Available data on the effects of anti-diabetic drugs on fracture risk are contradictory. Therefore, our study aimed to analyze all available data on the effects of anti-diabetic drugs on fracture risk in type 2 diabetes mellitus (T2DM) patients.

**Methods:**

Embase, Medline, ClinicalTrials.gov, and Cochrane CENTRAL were searched for relevant trials. All data analyses were performed with STATA (12.0) and R language (3.6.0). Risk ratio (RR) with its 95% confidence interval (CI) was calculated by combining data for the fracture effects of anti-diabetic drugs, including sodium–glucose co-transporter 2 (SGLT2) inhibitors, dipeptidyl peptidase-4 (DPP-4) inhibitors, glucagon-like peptide-1 (GLP-1) receptor agonists, meglitinides, α-glucosidase inhibitors, thiazolidinediones, biguanides, insulin, and sulfonylureas.

**Results:**

One hundred seventeen eligible randomized controlled trials (RCTs) with 221,364 participants were included in this study. Compared with placebo, trelagliptin (RR 3.51; 1.58–13.70) increased the risk of fracture, whereas albiglutide (RR 0.29; 0.04–0.93) and voglibose (RR 0.03; 0–0.11) decreased the risk of fracture. Other medications were comparable in terms of their effects on fracture risk, and no statistical significance was observed. In terms of fractures, voglibose (0.01%) may be the safest option, and trelagliptin (13.64%) may be the worst. Sensitivity analysis results were consistent with those of the main analysis. No statistically significant differences were observed in the regression coefficients of age (1.03; 0.32–2.1), follow-up duration (0.79; 0.27–1.64), and sex distribution (0.63; 0.15–1.56).

**Conclusions:**

We found varied results on the association between the use of anti-diabetic drugs and fracture risk. Specifically, trelagliptin raised the risk of fracture, whereas voglibose and albiglutide showed benefit with statistical difference. Other drugs were comparable in terms of their effects on fracture risk. Some drugs (omarigliptin, sitagliptin, vildagliptin, saxagliptin, empagliflozin, ertugliflozin, rosiglitazone, pioglitazone, and nateglinide) may increase the risk of fracture, while others (such as dulaglutide, exenatide, liraglutide, semaglutide, lixisenatide, linagliptin, alogliptin, canagliflozin, dapagliflozin, glipizide, gliclazide, glibenclamide, glimepiride, metformin, and insulin) may show benefits. The risk of fracture was independent of age, sex distribution, and the duration of exposure to anti-diabetic drugs. When developing individualized treatment strategies, the clinical efficacy of anti-diabetic drugs must be weighed against their benefits and risks brought about by individual differences of patients.

**Systematic Review Registration:**

This Systematic Review was prospectively registered on the PROSPERO (https://www.crd.york.ac.uk/PROSPERO/, registration number CRD42020189464).

## Introduction

Diabetes is a major global health problem. It affects nearly half a billion patients worldwide. Among diabetic patients, 90% suffer from type 2 diabetes mellitus (T2DM) (1, 2). Mounting evidence indicates that T2DM patients are at a higher risk of developing fragility fractures because their bone microenvironment is deteriorated by the disease (3, 4). T2DM patients with an increased bone mineral density (BMD) may suffer more from bone fractures. Many studies have suggested that a deteriorated bone quality, rather than a decreased BMD, may be the key factor influencing bone fragility in T2DM patients. From the perspective of clinical diagnosis, T2DM-related complications (for instance, neuropathy, macroangiopathy, and retinopathy) can be regarded as predictors of bone fractures, and drug therapies may have negative effects on bone quality (5). However, it is still not entirely clear why diabetes complications can lead to fragility fractures (6). Some studies have indicated that several mechanisms may be used to explain why patients with T2DM are more susceptible to fragility fractures, including oxidative stress, hyperglycemia, levels of insulin, risk of falls, functions of osteocalcin and adiponectin, variations in BMD, and treatment-induced hypoglycemia, all of which increase fracture risk in patients with T2DM (3, 6, 7). The fragility fractures caused by diabetes are fatally serious. They may require surgeries and may further develop into disabilities, paralysis, or deaths (8, 9). Therefore, the developed anti-diabetic treatment strategies should at least not increase the risk of bone fractures in the vulnerable population (10, 11).

Currently, multiple anti-diabetic drugs are available, but previous research did not integrate all related data into one analysis and compare the available anti-diabetic drugs head-to-head. Therefore, associations between fracture events and anti-diabetic drug effects have not been clearly elucidated (12–14). To address this problem, we herein utilized Bayesian meta-analysis, a validated and mature statistical method, to compare the effects of all available anti-diabetic drugs on fracture risk (15). This comprehensive review and meta-analysis aimed to evaluate the safety of anti-diabetic drugs in fracture events based on the data available from clinical trials. Our study may help clinical researchers investigate the risk of fracture related to the use of anti-diabetic drugs in future research.

## Methods

### Search Strategy

This study was prospectively registered on the PROSPERO (https://www.crd.york.ac.uk/PROSPERO/, registration number CRD42020189464). A search for “Anti-diabetic drug”, “Type 2 diabetes mellitus”, “thiazolidinediones”, “α-glucosidase”, “bromocriptine-QR”, “meglitinides”, “GLP-1 receptor agonists”, “biguanides”, “sulfonylureas”, “SGLT2 inhibitors”, “insulin”, and “DPP-4 inhibitors” was performed in Embase, Medline, ClinicalTrials.gov, and Cochrane CENTRAL to identify randomized controlled trials (RCTs) up to May 1, 2021, with English-language restriction.

### Selection Criteria

Clinical trials were eligible if they met the following criteria: 1) RCTs; 2) duration ≥12 months; 3) the intervention or comparators were with anti-diabetic drugs, including sulfonylureas, dipeptidyl peptidase-4 (DPP-4) inhibitors, bromocriptine-QR, meglitinides, sodium–glucose co-transporter 2 (SGLT2) inhibitors, thiazolidinediones, biguanides, glucagon-like peptide-1 (GLP-1) receptor agonists, insulin, α-glucosidase, and placebo; 4) data on fracture were available.

### Data Extraction and Quality Assessment

For the eligible studies, data were extracted by two reviewers (Y-SZ and YY) independently; the disagreements were resolved by two reviewers and, if necessary, consulted by a senior reviewer (B-CX). Cochrane risk-of-bias tool was used to estimate the risk of bias for eligible studies (16). The data on trials available, consisting of the first author, sample size, mean age, follow-up, intervention and comparators, HbA1c, and outcomes of interest, were extracted. Grading of Recommendations, Assessment, Development and Evaluation (GRADE) was performed to assess the quality of evidence for fracture outcomes included. The GRADE approach categorizes evidence into high, moderate, low, or very low quality.

### Data Analysis

The Bayesian meta-analysis model was established by performing the Markov chain Monte Carlo methods (17). Random-effects model was used to account for heterogeneity between clinical trials for Bayesian analysis model, risk ratios (RRs) with its 95% confidence interval (CI) of anti-diabetic drugs on bone fracture were evaluated, RR value <1 favors “lower risk”, RR value >1 favors “higher risk,” and it permits all comparisons (direct/indirect comparisons) to be taken into calculating synchronously (18, 19). The posterior distributions of the parameters model were generated by four chains (100,000 per chain, 400,000 iterations) in the random-effects model (20). We checked heterogeneity by performing the I^2^ statistic and verified the model fit by calculating residual deviance. In addition, we calculated inconsistency of the direct and indirect comparisons by operating node-splitting method, and p-value <0.05 was defined as inconsistency. We calculated rank treatment of each anti-diabetic drug to estimate the safest probability. In addition, we calculated meta-regression analysis to discover the association with the fracture risk and age, fracture risk and sex distribution, and fracture risk and length of duration (21); and we performed sensitivity analysis to detect the influence of data (22). A comparison-adjusted funnel plot was drawn by using STATA software to analyze publication bias (23). All data analyses were performed with R language (3.6.0) (24).

## Result

### Study Characteristics and Quality

**Figure 1:**
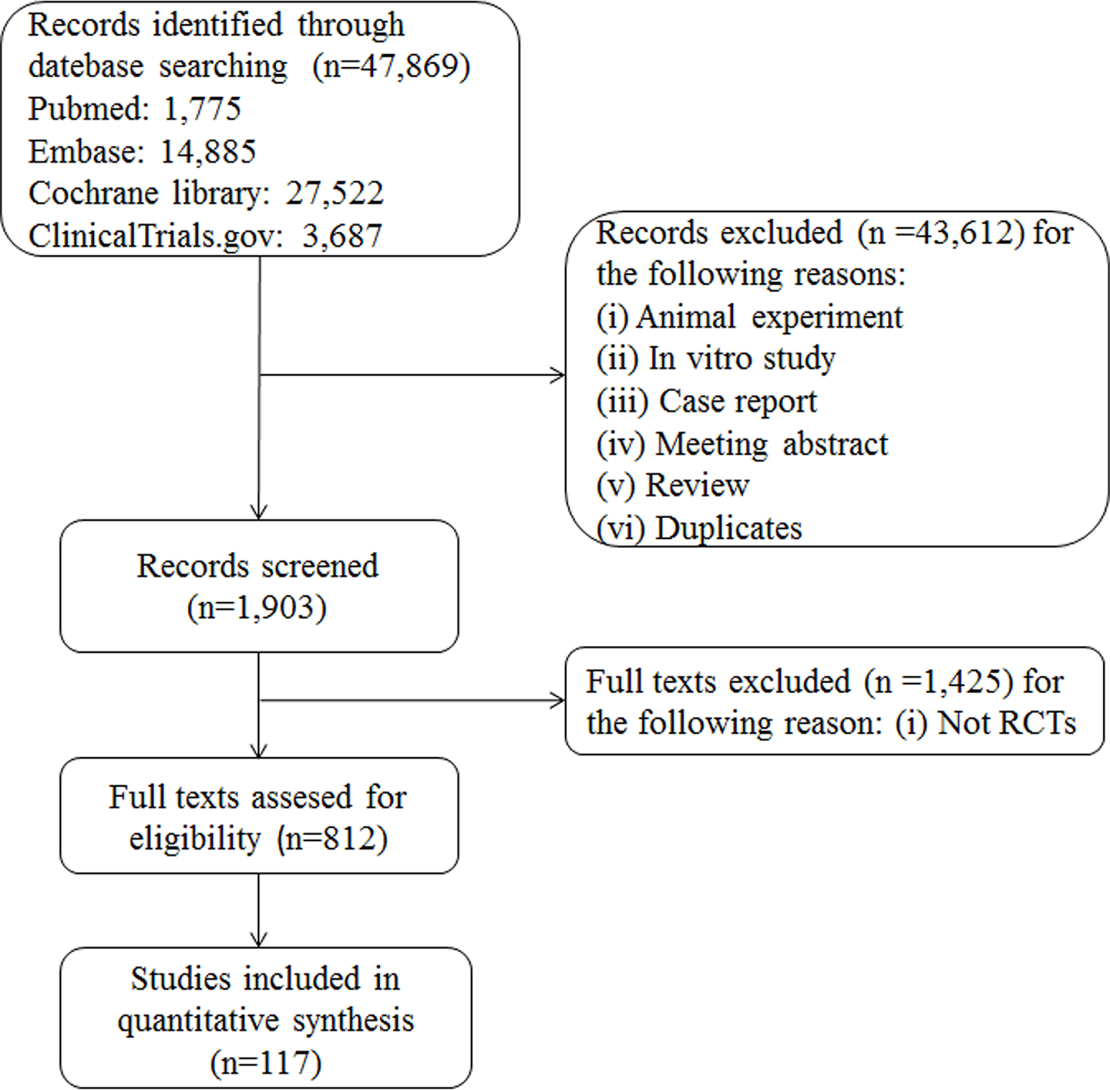
Trial flow diagram.

**Figure 2:**
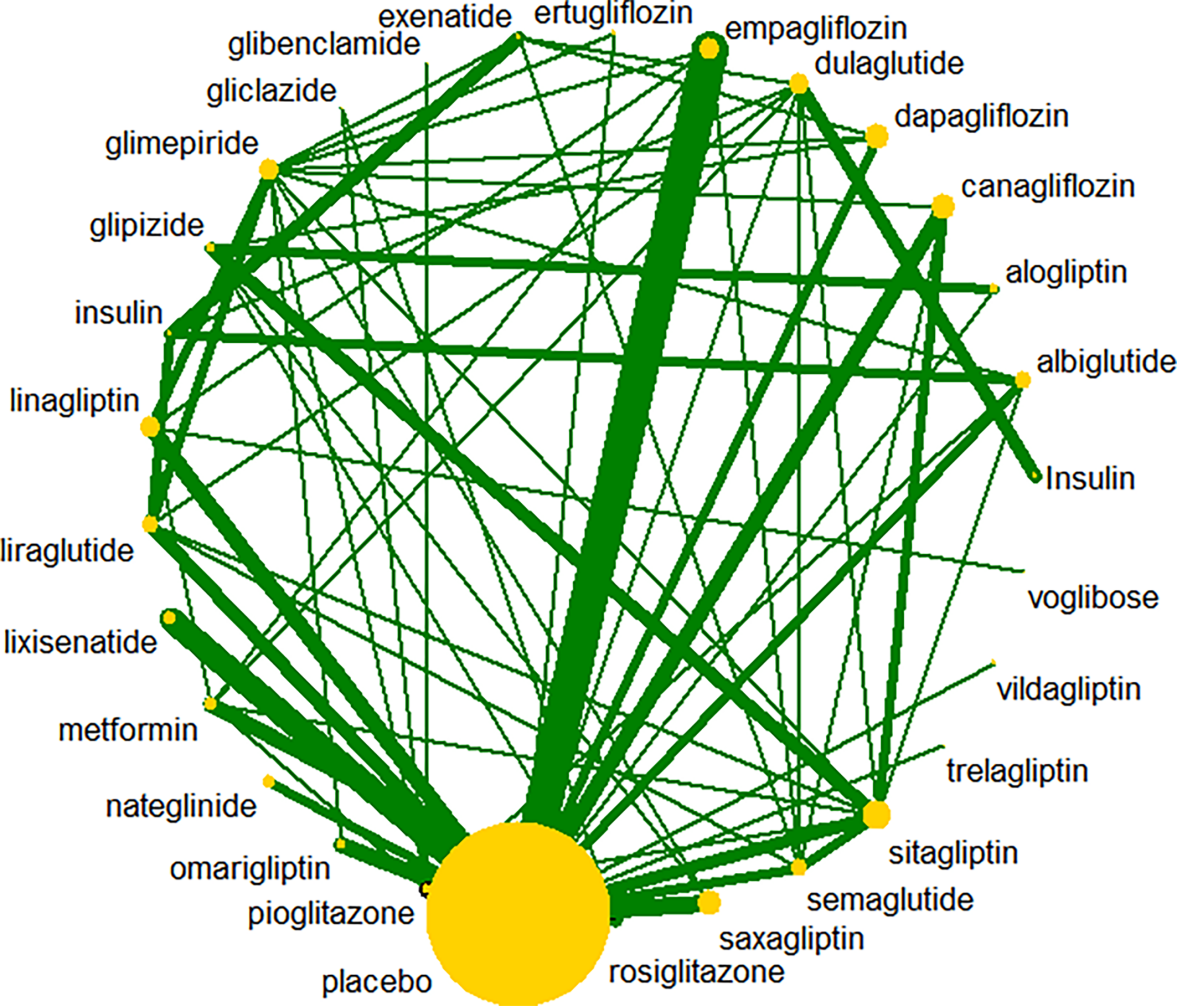
Network plot for the Bayesian network meta-analyses.

A total of 47,869 records were retrieved; after review of 812 records for eligibility, 117 RCTs were included. The interventions evaluated in the meta-analyses included nine types of anti-diabetic drugs: SGLT2 inhibitors, DPP-4 inhibitors, α-glucosidase inhibitors, thiazolidinediones, insulin, GLP-1 receptor agonists, meglitinides, biguanides, and sulfonylureas. The flowchart for selection of clinical trials is shown in **Figure 1**. All anti-diabetic drugs were connected to draw a network plot (**Figure 2**). Characteristics of the clinical trials with their quality analyses are shown in **Supplementary Tables 1**, **2**.

### Statistical Analysis

The model fit calculated by residual deviance was agreeable (ratio 1.148, I^2^ = 15%). The results of RRs are summarized in **Table 1**. The GRADE of quality evidence for anti-diabetic drugs on fracture outcomes is summarized in **Table 2**; all anti-diabetic drugs were graded as high/moderate quality in the present study.

**Table 1:** Estimated relative treatment effects as risk ratios (RRs) and its corresponding 95% confidence intervals (CIs).

Treatment	albiglutide	dulaglutide	exenatide	lixisenatide	liraglutide	semaglutide	vildagliptin	omarigliptin	trelagliptin	saxagliptin	alogliptin	sitagliptin	linagliptin	dapagliflozin	ertugliflozin	canagliflozin	empagliflozin	glipizide	glimepiride	glibenclamide	gliclazide	pioglitazone	rosiglitazone	nateglinide	metformin	insulin	voglibose	placebo
albiglutide	NA	0.43 (0.07, 2.49)	0.41 (0.06, 2.83)	0.36 (0.05, 2.11)	0.53 (0.08, 3.14)	0.59 (0.1, 3.41)	0.34 (0.05, 2.04)	0.29 (0.04, 2.05)	0.01 (0, 0.04)	0.19 (0.03, 1.15)	0.52 (0.07, 3.52)	0.3 (0.05, 1.65)	0.43 (0.07, 2.42)	0.43 (0.07, 2.67)	0.16 (0, 2.6)	0.64 (0.11, 3.45)	0.33 (0.06, 1.79)	0.58 (0.09, 3.73)	0.86 (0.15, 4.75)	0.4 (0.08, 1.92)	0.52 (0.03, 9.09)	0.34 (0.06, 1.92)	0.32 (0.05, 2.15)	0.29 (0.04, 1.84)	0.48 (0.07, 3.21)	0.58 (0.09, 3.76)	1.12 (1.01, 2.56)	0.29 (0.04, 0.93)
dulaglutide	2.33 (0.4, 14.65)	NA	0.96 (0.29, 3.19)	0.83 (0.25, 2.62)	1.24 (0.46, 3.3)	1.38 (0.51, 3.85)	0.78 (0.26, 2.29)	0.69 (0.18, 2.6)	0.01 (0, 0.05)	0.45 (0.13, 1.39)	1.2 (0.32, 4.52)	0.7 (0.28, 1.78)	1.01 (0.36, 2.79)	1.01 (0.32, 3.19)	0.38 (0.01, 1.05)	1.48 (0.55, 3.94)	0.77 (0.28, 2.06)	1.35 (0.42, 4.48)	2 (0.77, 5.36)	0.93 (0.42, 2.06)	1.22 (0.12, 15.06)	0.79 (0.29, 2.22)	0.76 (0.22, 2.64)	0.68 (0.2, 2.25)	1.12 (0.34, 3.86)	1.34 (0.55, 3.39)	1.91 (1.09, 7.52)	0.91 (0.17, 4.88)
exenatide	2.44 (0.35, 17.87)	1.04 (0.31, 3.46)	NA	0.86 (0.21, 3.41)	1.29 (0.36, 4.69)	1.43 (0.41, 5.11)	0.81 (0.21, 3.06)	0.72 (0.15, 3.34)	0.01 (0, 0.05)	0.46 (0.11, 1.86)	1.25 (0.27, 5.82)	0.73 (0.21, 2.56)	1.05 (0.3, 3.64)	1.05 (0.28, 3.94)	0.39 (0.01, 1.78)	1.54 (0.43, 5.34)	0.8 (0.23, 2.79)	1.41 (0.35, 5.86)	2.09 (0.68, 6.55)	0.97 (0.32, 2.97)	1.26 (0.11, 18.2)	0.82 (0.23, 3.02)	0.79 (0.18, 3.52)	0.7 (0.16, 2.99)	1.16 (0.27, 5.18)	1.39 (0.43, 4.67)	2.05 (1.16, 7.44)	0.95 (0.15, 5.96)
lixisenatide	2.81 (0.47, 18.77)	1.21 (0.38, 4)	1.16 (0.29, 4.84)	NA	1.5 (0.48, 4.93)	1.67 (0.55, 5.31)	0.95 (0.29, 3.25)	0.83 (0.21, 3.39)	0.02 (0, 0.06)	0.54 (0.15, 1.83)	1.45 (0.37, 6.02)	0.85 (0.29, 2.53)	1.22 (0.41, 3.71)	1.22 (0.36, 4.39)	0.45 (0.01, 1.24)	1.79 (0.63, 5.22)	0.92 (0.33, 2.72)	1.63 (0.48, 6.04)	2.41 (0.86, 7.45)	1.12 (0.49, 2.74)	1.48 (0.14, 19.54)	0.96 (0.33, 2.97)	0.92 (0.25, 3.58)	0.81 (0.25, 2.92)	1.35 (0.37, 5.33)	1.63 (0.46, 6.21)	2.33 (1.99, 6.09)	0.9 (0.2, 6.3)
liraglutide	1.87 (0.32, 11.82)	0.8 (0.3, 2.17)	0.78 (0.21, 2.8)	0.67 (0.2, 2.1)	NA	1.11 (0.41, 3.06)	0.63 (0.2, 1.93)	0.55 (0.14, 2.13)	0.01 (0, 0.04)	0.36 (0.11, 1.14)	0.97 (0.26, 3.72)	0.57 (0.22, 1.48)	0.81 (0.29, 2.25)	0.81 (0.25, 2.66)	0.3 (0.01, 1.03)	1.19 (0.45, 3.18)	0.61 (0.23, 1.66)	1.09 (0.33, 3.69)	1.6 (0.64, 4.22)	0.74 (0.34, 1.67)	0.97 (0.1, 12.74)	0.64 (0.23, 1.83)	0.61 (0.17, 2.19)	0.54 (0.17, 1.83)	0.9 (0.26, 3.22)	1.08 (0.36, 3.33)	1.43 (1.07, 3.24)	0.73 (0.14, 3.92)
semaglutide	1.69 (0.29, 10.48)	0.73 (0.26, 1.98)	0.7 (0.2, 2.45)	0.6 (0.19, 1.81)	0.9 (0.33, 2.44)	NA	0.57 (0.18, 1.72)	0.5 (0.13, 1.84)	0.01 (0, 0.03)	0.32 (0.1, 0.99)	0.88 (0.23, 3.13)	0.51 (0.2, 1.24)	0.73 (0.26, 1.94)	0.73 (0.23, 2.32)	0.27 (0.01, 0.96)	1.07 (0.42, 2.66)	0.56 (0.22, 1.41)	0.99 (0.31, 3.17)	1.45 (0.57, 3.78)	0.67 (0.32, 1.42)	0.88 (0.09, 11.16)	0.58 (0.21, 1.56)	0.55 (0.16, 1.93)	0.49 (0.15, 1.59)	0.81 (0.24, 2.81)	0.98 (0.31, 3.17)	1.35 (1.06, 3.85)	0.66 (0.13, 3.41)
vildagliptin	2.97 (0.49, 19.28)	1.28 (0.44, 3.8)	1.23 (0.33, 4.67)	1.05 (0.31, 3.51)	1.58 (0.52, 4.91)	1.76 (0.58, 5.47)	NA	0.88 (0.22, 3.52)	0.02 (0, 0.06)	0.57 (0.16, 1.92)	1.53 (0.38, 6.17)	0.9 (0.33, 2.5)	1.29 (0.44, 3.77)	1.3 (0.38, 4.37)	0.48 (0.01, 1.35)	1.88 (0.67, 5.41)	0.98 (0.35, 2.81)	1.74 (0.5, 6.15)	2.56 (0.96, 7.23)	1.18 (0.51, 2.87)	1.55 (0.15, 20.06)	1.01 (0.38, 2.85)	0.97 (0.27, 3.67)	0.86 (0.25, 3.06)	1.43 (0.4, 5.39)	1.72 (0.57, 5.43)	2.12 (1.13, 6.07)	1.17 (0.23, 6.16)
omarigliptin	3.4 (0.49, 24.58)	1.45 (0.38, 5.48)	1.39 (0.3, 6.51)	1.2 (0.29, 4.7)	1.81 (0.47, 6.92)	2 (0.54, 7.43)	1.14 (0.28, 4.46)	NA	0.02 (0, 0.07)	0.65 (0.15, 2.57)	1.74 (0.38, 8.18)	1.03 (0.29, 3.64)	1.47 (0.41, 5.28)	1.47 (0.36, 5.92)	0.54 (0.02, 1.77)	2.15 (0.62, 7.53)	1.11 (0.32, 3.89)	1.97 (0.48, 8.4)	2.9 (0.86, 10.13)	1.34 (0.46, 4.07)	1.76 (0.15, 25.3)	1.16 (0.32, 4.13)	1.11 (0.24, 4.85)	0.98 (0.24, 4.1)	1.63 (0.37, 7.2)	1.95 (0.46, 8.4)	2.80 (1.28, 8.02)	1.33 (0.21, 8.24)
trelagliptin	4.9 (2.32, 19.08)	5.60 (2.24, 14.25)	5.28 (1.41, 13.61)	4.62 (1.43, 13.19)	3.71 (1.66, 14.84)	5.76 (2.08, 19.37)	4.39 (1.09, 13.25)	3.76 (1.28, 11.43)	NA	2.588 (1.89, 6.84)	4.60 (2.41, 16.63)	3.95 (1.8, 12.84)	5.72 (2.61, 22.46)	5.82 (2.81, 14.09)	3.71 (1.82, 10.4)	4.83 (2.8, 18.06)	4.46 (1.57, 12.97)	5.77 (2.93, 25.60)	8.22 (4.85, 29.1)	3.62 (1.63, 14.85)	6.67 (3.43, 29.04)	4.44 (1.74, 14.92)	4.34 (1.48, 13.05)	3.88 (1.48, 12.97)	4.63 (2.53, 14.47)	6.72 (2.5, 25.69)	7.43 (2.53, 22.23)	3.51 (1.58, 13.70)
saxagliptin	5.28 (0.87, 35.44)	2.25 (0.72, 7.64)	2.18 (0.54, 9.21)	1.87 (0.55, 6.55)	2.78 (0.87, 9.49)	3.09 (1.01, 10.46)	1.75 (0.52, 6.38)	1.54 (0.39, 6.58)	0.03 (0, 0.11)	NA	2.71 (0.72, 10.84)	1.58 (0.56, 4.8)	2.26 (0.78, 7.15)	2.27 (0.69, 8.19)	0.85 (0.03, 2.82)	3.32 (1.17, 10.23)	1.72 (0.6, 5.3)	3.05 (0.98, 10.31)	4.5 (1.65, 13.89)	2.08 (0.88, 5.48)	2.75 (0.27, 37.1)	1.78 (0.61, 5.76)	1.7 (0.47, 6.86)	1.52 (0.45, 5.75)	2.5 (0.7, 10.16)	3.03 (0.85, 12.4)	2.21 (1.29, 6.86)	2.04 (0.38, 12.09)
alogliptin	1.94 (0.28, 14.28)	0.84 (0.22, 3.13)	0.8 (0.17, 3.75)	0.69 (0.17, 2.72)	1.03 (0.27, 3.91)	1.14 (0.32, 4.34)	0.65 (0.16, 2.6)	0.57 (0.12, 2.66)	0.01 (0, 0.04)	0.37 (0.09, 1.4)	NA	0.58 (0.18, 1.94)	0.84 (0.23, 3)	0.84 (0.22, 3.31)	0.31 (0.01, 1.83)	1.23 (0.35, 4.23)	0.64 (0.18, 2.23)	1.13 (0.38, 3.46)	1.67 (0.49, 5.93)	0.77 (0.26, 2.32)	1.01 (0.09, 14.19)	0.66 (0.19, 2.4)	0.64 (0.15, 2.71)	0.56 (0.14, 2.36)	0.93 (0.22, 3.99)	1.13 (0.26, 4.91)	1.39 (1.02, 6.18)	0.76 (0.12, 4.87)
sitagliptin	3.29 (0.6, 19.83)	1.42 (0.56, 3.61)	1.37 (0.39, 4.73)	1.17 (0.4, 3.4)	1.77 (0.68, 4.65)	1.96 (0.8, 4.9)	1.11 (0.4, 3.06)	0.97 (0.27, 3.5)	0.02 (0, 0.07)	0.63 (0.21, 1.79)	1.71 (0.51, 5.68)	NA	1.43 (0.57, 3.62)	1.44 (0.5, 4.22)	0.54 (0.02, 1.58)	2.1 (0.91, 4.85)	1.09 (0.45, 2.63)	1.92 (0.75, 5.12)	2.84 (1.22, 6.98)	1.32 (0.69, 2.58)	1.73 (0.18, 21.35)	1.12 (0.46, 2.82)	1.08 (0.35, 3.37)	0.96 (0.32, 3.01)	1.59 (0.53, 4.98)	1.91 (0.63, 5.91)	2.69 (1.28, 9.04)	1.29 (0.27, 6.47)
linagliptin	2.33 (0.41, 14.11)	0.99 (0.36, 2.79)	0.95 (0.27, 3.29)	0.82 (0.27, 2.45)	1.24 (0.45, 3.4)	1.37 (0.52, 3.78)	0.78 (0.27, 2.27)	0.68 (0.19, 2.45)	0.01 (0, 0.05)	0.44 (0.14, 1.29)	1.19 (0.33, 4.35)	0.7 (0.28, 1.76)	NA	1 (0.34, 3.06)	0.38 (0.01, 1.38)	1.47 (0.59, 3.63)	0.76 (0.32, 1.85)	1.34 (0.44, 4.3)	1.99 (0.97, 4.25)	0.92 (0.46, 1.88)	1.21 (0.12, 14.75)	0.79 (0.31, 2.06)	0.75 (0.23, 2.53)	0.67 (0.22, 2.11)	1.11 (0.36, 3.66)	1.34 (0.43, 4.4)	2.04 (1.09, 3.254)	0.9 (0.18, 4.66)
dapagliflozin	2.31 (0.38, 15.1)	0.99 (0.31, 3.16)	0.95 (0.25, 3.53)	0.82 (0.23, 2.74)	1.23 (0.38, 3.97)	1.36 (0.43, 4.31)	0.77 (0.23, 2.65)	0.68 (0.17, 2.74)	0.01 (0, 0.05)	0.44 (0.12, 1.46)	1.19 (0.3, 4.59)	0.69 (0.24, 2.02)	1 (0.33, 2.98)	NA	0.37 (0.01, 1.32)	1.46 (0.5, 4.28)	0.76 (0.26, 2.24)	1.35 (0.42, 4.31)	1.98 (0.73, 5.63)	0.91 (0.38, 2.28)	1.2 (0.11, 15.72)	0.78 (0.26, 2.4)	0.75 (0.2, 2.89)	0.67 (0.19, 2.42)	1.11 (0.3, 4.31)	1.32 (0.38, 4.89)	1.96 (1.37, 6.13)	0.9 (0.16, 5.14)
ertugliflozin	6.42 (0.38, 16.51)	2.66 (0.25, 8.78)	2.58 (0.21, 9.27)	2.21 (0.19, 7.26)	3.33 (0.3, 7.35)	3.68 (0.34, 6.2)	2.07 (0.19, 6.61)	1.85 (0.15, 4.93)	0.04 (0, 0.17)	1.18 (0.1, 8.95)	3.25 (0.26, 7.85)	1.87 (0.18, 5.42)	2.66 (0.26, 8.2)	2.72 (0.24, 8.63)	NA	3.92 (0.38, 12.78)	2.04 (0.2, 6.22)	3.65 (0.32, 12.41)	5.29 (0.6, 16.59)	2.45 (0.26, 7.53)	3.44 (0.14, 9.5)	2.13 (0.2, 6.24)	2.06 (0.17, 7.17)	1.81 (0.16, 6.16)	3.02 (0.25, 6.06)	3.61 (0.3, 11.97)	6.13 (2.37, 19.91)	2.47 (0.16, 9.95)
canagliflozin	1.57 (0.29, 9.22)	0.68 (0.25, 1.82)	0.65 (0.19, 2.3)	0.56 (0.19, 1.58)	0.84 (0.31, 2.24)	0.93 (0.38, 2.36)	0.53 (0.18, 1.49)	0.47 (0.13, 1.62)	0.01 (0, 0.03)	0.3 (0.1, 0.86)	0.81 (0.24, 2.82)	0.48 (0.21, 1.1)	0.68 (0.28, 1.69)	0.69 (0.23, 2.02)	0.26 (0.01, 0.65)	NA	0.52 (0.22, 1.23)	0.92 (0.32, 2.8)	1.35 (0.6, 3.2)	0.63 (0.34, 1.19)	0.82 (0.09, 10.05)	0.54 (0.22, 1.37)	0.51 (0.16, 1.71)	0.46 (0.16, 1.38)	0.76 (0.24, 2.5)	0.91 (0.3, 2.9)	1.32 (1.07, 3.64)	0.62 (0.13, 3.08)
empagliflozin	3.03 (0.56, 18)	1.31 (0.49, 3.51)	1.26 (0.36, 4.35)	1.08 (0.37, 3.05)	1.63 (0.6, 4.34)	1.79 (0.71, 4.63)	1.02 (0.36, 2.89)	0.9 (0.26, 3.13)	0.12 (0, 0.36)	0.58 (0.19, 1.67)	1.56 (0.45, 5.47)	0.92 (0.38, 2.21)	1.31 (0.54, 3.15)	1.32 (0.45, 3.83)	0.49 (0.02, 1.02)	1.93 (0.82, 4.55)	NA	1.77 (0.59, 5.4)	2.61 (1.17, 6.02)	1.21 (0.66, 2.29)	1.58 (0.16, 19.15)	1.04 (0.42, 2.61)	0.99 (0.31, 3.2)	0.88 (0.3, 2.67)	1.46 (0.47, 4.68)	1.76 (0.57, 5.56)	2.64 (1.83, 8.36)	1.19 (0.24, 5.89)
glipizide	1.72 (0.27, 11.65)	0.74 (0.22, 2.38)	0.71 (0.17, 2.89)	0.61 (0.17, 2.1)	0.92 (0.27, 3)	1.01 (0.32, 3.24)	0.57 (0.16, 2.01)	0.51 (0.12, 2.09)	0.01 (0, 0.04)	0.33 (0.1, 1.02)	0.89 (0.29, 2.64)	0.52 (0.2, 1.33)	0.74 (0.23, 2.26)	0.74 (0.23, 2.38)	0.27 (0.01, 0.82)	1.09 (0.36, 3.17)	0.57 (0.19, 1.69)	NA	1.48 (0.5, 4.43)	0.68 (0.27, 1.72)	0.89 (0.08, 11.76)	0.59 (0.18, 1.82)	0.56 (0.16, 1.98)	0.5 (0.14, 1.83)	0.82 (0.23, 2.92)	0.99 (0.26, 3.73)	1.37 (1.10, 3.87)	0.67 (0.12, 3.74)
glimepiride	1.16 (0.21, 6.71)	0.5 (0.19, 1.29)	0.48 (0.15, 1.47)	0.41 (0.13, 1.17)	0.62 (0.24, 1.55)	0.69 (0.26, 1.76)	0.39 (0.14, 1.05)	0.34 (0.1, 1.16)	0.01 (0, 0.02)	0.22 (0.07, 0.61)	0.6 (0.17, 2.06)	0.35 (0.14, 0.82)	0.5 (0.24, 1.03)	0.51 (0.18, 1.38)	0.19 (0.01, 0.71)	0.74 (0.31, 1.66)	0.38 (0.17, 0.85)	0.68 (0.23, 2.02)	NA	0.46 (0.24, 0.87)	0.61 (0.06, 7.19)	0.4 (0.16, 0.94)	0.38 (0.12, 1.23)	0.34 (0.11, 1.01)	0.56 (0.18, 1.81)	0.67 (0.22, 1.99)	1.12 (1.04, 2.96)	0.45 (0.09, 2.17)
glibenclamide	2.51 (0.52, 13.15)	1.08 (0.48, 2.37)	1.03 (0.34, 3.17)	0.89 (0.37, 2.05)	1.34 (0.6, 2.92)	1.49 (0.71, 3.12)	0.85 (0.35, 1.97)	0.74 (0.25, 2.18)	0.01 (0, 0.03)	0.48 (0.18, 1.13)	1.29 (0.43, 3.82)	0.76 (0.39, 1.46)	1.09 (0.53, 2.16)	1.09 (0.44, 2.65)	0.41 (0.01, 1.32)	1.59 (0.84, 2.91)	0.83 (0.44, 1.52)	1.46 (0.58, 3.77)	2.16 (1.15, 4.19)	NA	1.31 (0.15, 14.9)	0.86 (0.43, 1.7)	0.82 (0.29, 2.3)	0.73 (0.3, 1.78)	1.2 (0.44, 3.39)	1.45 (0.55, 3.91)	7.51 (2.10, 25.51)	0.98 (0.22, 4.32)
gliclazide	1.91 (0.11, 30.33)	0.82 (0.07, 8.37)	0.79 (0.05, 9.35)	0.68 (0.05, 7.16)	1.03 (0.08, 10.26)	1.14 (0.09, 11.34)	0.64 (0.05, 6.52)	0.57 (0.04, 6.47)	0.01 (0, 0.04)	0.36 (0.03, 3.75)	0.99 (0.07, 11.22)	0.58 (0.05, 5.53)	0.82 (0.07, 8.15)	0.83 (0.06, 8.84)	0.29 (0.01, 0.73)	1.21 (0.1, 11.71)	0.63 (0.05, 6.16)	1.12 (0.09, 11.91)	1.65 (0.14, 15.9)	0.76 (0.07, 6.89)	NA	0.66 (0.06, 5.7)	0.63 (0.05, 5.83)	0.56 (0.04, 5.99)	0.93 (0.08, 8.88)	1.1 (0.08, 11.96)	1.49 (1.22, 4.07)	0.75 (0.05, 9.46)
pioglitazone	2.93 (0.52, 17.49)	1.26 (0.45, 3.46)	1.21 (0.33, 4.35)	1.04 (0.34, 3.07)	1.57 (0.55, 4.32)	1.73 (0.64, 4.72)	0.99 (0.35, 2.64)	0.86 (0.24, 3.08)	0.02 (0, 0.06)	0.56 (0.17, 1.65)	1.51 (0.42, 5.36)	0.89 (0.35, 2.15)	1.27 (0.49, 3.23)	1.28 (0.42, 3.84)	0.47 (0.01, 1.14)	1.86 (0.73, 4.58)	0.96 (0.38, 2.39)	1.71 (0.55, 5.42)	2.52 (1.06, 6.11)	1.17 (0.59, 2.32)	1.52 (0.18, 16.92)	NA	0.96 (0.3, 3.05)	0.85 (0.27, 2.64)	1.41 (0.45, 4.46)	1.69 (0.54, 5.41)	2.15 (1.17, 6.33)	1.14 (0.31, 4.25)
rosiglitazone	3.08 (0.47, 21.18)	1.32 (0.38, 4.46)	1.27 (0.28, 5.55)	1.09 (0.28, 4.06)	1.64 (0.46, 5.87)	1.83 (0.52, 6.27)	1.03 (0.27, 3.75)	0.9 (0.21, 4.09)	0.02 (0, 0.06)	0.59 (0.15, 2.15)	1.57 (0.37, 6.77)	0.93 (0.3, 2.85)	1.33 (0.39, 4.37)	1.34 (0.35, 5.06)	0.49 (0.01, 1.79)	1.94 (0.59, 6.26)	1.01 (0.31, 3.21)	1.79 (0.5, 6.41)	2.64 (0.81, 8.65)	1.22 (0.43, 3.42)	1.59 (0.17, 18.42)	1.04 (0.33, 3.35)	NA	0.89 (0.23, 3.48)	1.48 (0.75, 2.89)	1.77 (0.46, 7.06)	2.47 (1.91, 8.73)	1.2 (0.21, 6.83)
nateglinide	3.46 (0.54, 22.29)	1.48 (0.44, 4.89)	1.42 (0.33, 6.07)	1.23 (0.34, 4.08)	1.85 (0.55, 5.93)	2.04 (0.63, 6.53)	1.16 (0.33, 3.97)	1.02 (0.24, 4.21)	0.02 (0, 0.07)	0.66 (0.17, 2.23)	1.78 (0.42, 7.23)	1.04 (0.33, 3.13)	1.49 (0.47, 4.63)	1.5 (0.41, 5.25)	0.55 (0.02, 1.24)	2.19 (0.73, 6.38)	1.13 (0.37, 3.34)	2.01 (0.55, 7.32)	2.95 (0.99, 9.15)	1.37 (0.56, 3.34)	1.79 (0.17, 23.68)	1.18 (0.38, 3.64)	1.12 (0.29, 4.36)	NA	1.66 (0.43, 6.51)	2 (0.53, 7.56)	2.71 (1.31, 7.34)	1.35 (0.24, 7.55)
metformin	2.08 (0.31, 14.28)	0.89 (0.26, 2.94)	0.86 (0.19, 3.67)	0.74 (0.19, 2.69)	1.11 (0.31, 3.91)	1.23 (0.36, 4.18)	0.7 (0.19, 2.49)	0.61 (0.14, 2.7)	0.01 (0, 0.04)	0.4 (0.1, 1.43)	1.07 (0.25, 4.51)	0.63 (0.2, 1.88)	0.9 (0.27, 2.8)	0.9 (0.23, 3.34)	0.33 (0.01, 0.94)	1.32 (0.4, 4.15)	0.69 (0.21, 2.13)	1.22 (0.34, 4.3)	1.8 (0.55, 5.65)	0.83 (0.29, 2.27)	1.08 (0.11, 12.96)	0.71 (0.22, 2.22)	0.68 (0.35, 1.33)	0.6 (0.15, 2.32)	NA	1.2 (0.31, 4.73)	1.40 (7.89, 4.51)	0.81 (0.14, 4.56)
insulin	1.74 (0.27, 11.65)	0.75 (0.29, 1.8)	0.72 (0.21, 2.31)	0.61 (0.16, 2.19)	0.93 (0.3, 2.77)	1.02 (0.32, 3.27)	0.58 (0.18, 1.76)	0.51 (0.12, 2.16)	0.01 (0, 0.04)	0.33 (0.08, 1.18)	0.89 (0.2, 3.78)	0.52 (0.17, 1.58)	0.75 (0.23, 2.35)	0.76 (0.2, 2.66)	0.28 (0.01, 0.83)	1.1 (0.34, 3.38)	0.57 (0.18, 1.76)	1.01 (0.27, 3.78)	1.49 (0.5, 4.46)	0.69 (0.26, 1.82)	0.91 (0.08, 11.8)	0.59 (0.18, 1.87)	0.57 (0.14, 2.2)	0.5 (0.13, 1.89)	0.83 (0.21, 3.24)	NA	1.37 (1.16, 3.96)	0.68 (0.12, 3.86)
voglibose	0.08 (0, 0.28)	0.02 (0, 0.11)	0.02 (0, 0.11)	0.03 (0, 0.09)	0.03 (0, 0.14)	0.04 (0, 0.15)	0.03 (0, 0.09)	0.02 (0, 0.08)	0.01 (0, 0.03)	0.01 (0, 0.05)	0.03 (0, 0.14)	0.02 (0, 0.08)	0.03 (0, 0.11)	0.02 (0, 0.11)	0.01 (0, 0.05)	0.04 (0, 0.16)	0.02 (0, 0.08)	0.04 (0, 0.15)	0.07 (0, 0.23)	0.03 (0, 0.1)	0.04 (0, 0.15)	0.02 (0, 0.08)	0.02 (0, 0.08)	0.03 (0, 0.08)	0.04 (0, 0.13)	0.04 (0, 0.15)	NA	0.03 (0, 0.11)
placebo	2.57 (1.3, 9.22)	1.1 (0.2, 5.79)	1.06 (0.17, 6.6)	1.31 (0.16, 4.89)	1.37 (0.26, 7.1)	1.52 (0.29, 7.76)	0.86 (0.16, 4.44)	0.75 (0.12, 4.71)	0.01 (0, 0.05)	0.49 (0.08, 2.64)	1.32 (0.21, 8.12)	0.77 (0.15, 3.73)	1.11 (0.21, 5.47)	1.11 (0.19, 6.2)	0.4 (0.01, 1.67)	1.62 (0.32, 7.92)	0.84 (0.17, 4.13)	1.49 (0.27, 8.53)	2.2 (0.46, 10.81)	1.02 (0.23, 4.46)	1.33 (0.11, 20.63)	0.87 (0.24, 3.24)	0.83 (0.15, 4.81)	0.74 (0.13, 4.2)	1.23 (0.22, 7.06)	1.48 (0.26, 8.53)	7.38 (2.68, 24.04)	NA

Comparisons should be read from left to right. The estimate is located at the intersection of the treatments in the column heads and the treatments in the row heads. An RR value >1 favors the column-defining treatment. An RR value <1 favors the row-defining treatment; NA, not applicable.

**Table 2:** GRADE of quality evidence for glucose-lowering medications on fracture outcomes.

Medications	Risk of Bias	Inconsistency	Indirectness	Imprecision	Publication bias	Quality	Risk of fracture
Alogliptin	Not serious	Not serious	Undetected	Not serious	Not serious	High	Low
Linagliptin	Not serious	Serious	Undetected	Not serious	Not serious	Moderate	Low
Saxagliptin	Not serious	Not serious	Not serious	Not serious	Not serious	High	High
Sitagliptin	Not serious	Serious	Not serious	Not serious	Not serious	Moderate	High
Vildagliptin	Not serious	Not serious	Not serious	Not serious	Not serious	High	High
Omarigliptin	Not serious	Serious	Not serious	Not serious	Not serious	Moderate	High
Trelagliptin	Not serious	Not serious	Undetected	Not serious	Not serious	High	Very high
Albiglutide	Not serious	Not serious	Not serious	Not serious	Not serious	High	Very low
Dulaglutide	Not serious	Serious	Not serious	Not serious	Not serious	Moderate	Low
Exenatide	Not serious	Not serious	Not serious	Not serious	Not serious	High	Low
Liraglutide	Not serious	Serious	Not serious	Not serious	Not serious	Moderate	Low
Lixisenatide	Not serious	Not serious	Undetected	Not serious	Not serious	High	Low
Semaglutide	Not serious	Not serious	Not serious	Not serious	Not serious	High	Low
Canagliflozin	Not serious	Not serious	Not serious	Not serious	Not serious	High	Low
Dapagliflozin	Not serious	Not serious	Not serious	Not serious	Not serious	High	Low
Empagliflozin	Not serious	Not serious	Not serious	Not serious	Not serious	High	High
Ertugliflozin	Not serious	Serious	Not serious	Not serious	Not serious	Moderate	High
Glimepiride	Not serious	Not serious	Not serious	Not serious	Not serious	High	Low
Gliclazide	Not serious	Not serious	Undetected	Not serious	Not serious	High	Low
Glipizide	Not serious	Not serious	Not serious	Not serious	Not serious	High	Low
Rosiglitazone	Not serious	Not serious	Undetected	Not serious	Not serious	High	High
Pioglitazone	Not serious	Not serious	Not serious	Not serious	Not serious	High	High
Metformin	Not serious	Serious	Not serious	Not serious	Not serious	Moderate	Low
Voglibose	Not serious	Not serious	Undetected	Not serious	Not serious	High	Very low
Nateglinide	Not serious	Not serious	Undetected	Not serious	Not serious	High	High
Glibenclamide	Not serious	Not serious	Not serious	Not serious	Not serious	High	Low
Insulin	Not serious	Not serious	Not serious	Not serious	Not serious	High	Low

GRADE, Grading of Recommendations, Assessment, Development and Evaluation.

### Dipeptidyl Peptidase-4 Inhibitors

In the overall analysis, compared with placebo, we found varied results on the association between the use of DPP-4 inhibitors and fracture risk. Specifically, omarigliptin (RR 1.33; 0.21–8.24), sitagliptin (RR 1.29; 0.27–6.47), vildagliptin (RR 1.17; 0.23–6.16), and saxagliptin (RR 2.04; 0.38–12.09) raised the risk of fracture; whereas linagliptin (RR 0.9; 0.18–4.66) and alogliptin (RR 0.76; 0.12–4.87) reduced the risk. Additionally, trelagliptin (RR 3.51; 1.58–13.70) raised the risk of fracture with a statistical significance.

### Glucagon-Like Peptide-1 Receptor Agonists

We found that GLP-1 receptor agonists showed benefits as compared with placebo. The effects of dulaglutide (RR 0.91; 0.17–4.88), exenatide (RR 0.95; 0.15–5.96), liraglutide (RR 0.73; 0.14–3.92), semaglutide (RR 0.66; 95% 0.13–3.41), and lixisenatide (RR 0.92; 0.2–6.3) were comparable and showed no statistically significant differences. Additionally, albiglutide (RR 0.29; 0.04–0.93) showed benefits with a statistical significance.

### Sodium–Glucose Co-Transporter 2 Inhibitors

In the overall analysis, compared with placebo, canagliflozin (RR 0.62; 0.13–3.08) and dapagliflozin (RR 0.9; 0.16–5.14) decreased the risk of fracture; whereas empagliflozin (RR 1.19; 0.24–5.89) and ertugliflozin (RR 2.47; 95% 0.16–9.95) increased the risk of fracture, although the difference was not significant.

### Sulfonylureas

In the overall analysis, the results showed that glipizide (RR 0.67; 0.12–3.74), gliclazide (RR 0.75; 0.05–9.46), glibenclamide (RR 0.98; 0.22–4.25), and glimepiride (RR 0.45; 0.09–2.17) showed benefits as compared with placebo. Unfortunately, the differences were not statistically significant.

### Thiazolidinediones

In the overall analysis, the results suggest that rosiglitazone (RR 1.2; 0.21–6.83) and pioglitazone (RR 1.14; 0.31–4.25) increased the risk of facture as compared with placebo.

### Others

In the overall analysis, compared with placebo, the results suggested that metformin (RR 0.81; 0.14–4.56), voglibose (RR 0.03; 0–0.11), and insulin (RR 0.68; 0.12–3.86) showed benefit, whereas nateglinide (RR 1.35; 0.24–7.55) raised the risk of fracture.

### Ranking Probability

Based on surfaces under the cumulative probability cumulative ranking curves (SUCRAs), the probability ranking of anti-diabetic drugs is shown in **Supplementary Table 3**. In terms of the risk of inducing fracture, the safest treatment was voglibose (0.01%), and the worst treatment was trelagliptin (13.64%). According to GRADE, the quality of evidence for fracture outcomes was rated as high for most comparisons (**Table 2**). Quality of evidence was high for the overall ranking of anti-diabetic drug treatments.

### Heterogeneity and Inconsistence Check

Inconsistency was detected in some direct/indirect comparisons (**Supplementary Table 4**), in sitagliptin versus liraglutide, sitagliptin versus glimepiride, empagliflozin versus linagliptin, omarigliptin versus glimepiride, ertugliflozin versus glimepiride, dulaglutide versus metformin, omarigliptin versus glibenclamide, and ertugliflozin versus glibenclamide. The global heterogeneity was 44% calculated by R software (**Supplementary Table 5**); no statistically significant heterogeneity was detected in the direct/indirect comparisons.

### Funnel Plot and Publication Bias

**Figure 3:**
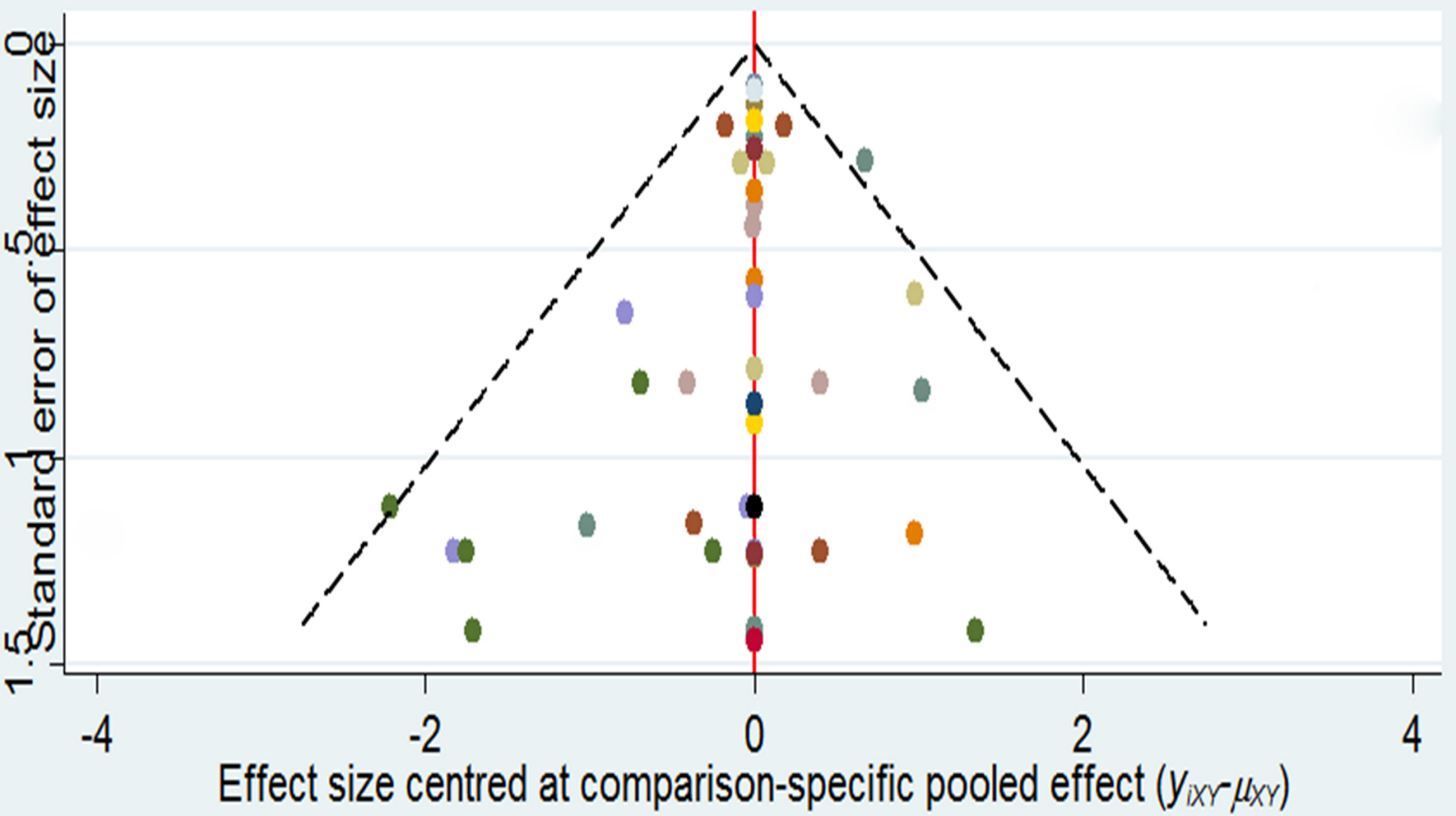
Comparison-adjusted funnel plots of the network.

As it is shown in **Figure 3**, it did not suggest any publication bias in the comparison-adjusted funnel plots.

### Sensitivity Analysis and Meta-Regression

Almost all the results of sensitivity analysis were consistent with those of the main analysis (**Supplementary Table 6**). No significant differences were observed in the regression coefficients (RCs). The risk of fracture was independent of age (RC 1.03; 0.32–2.1), duration of treatment (RC 0.79; 0.27–1.64), and sex distribution (RC 0.63; 0.15–1.56), but fracture risk had no clear associations with plasma glucose, level, and drug doses in patients treated with anti-diabetic drugs.

## Discussion

### Principal Findings

Previous studies have not fully demonstrated the effects of all anti-diabetic drugs on the risk of fracture in T2DM patients due to many limitations. One of the limitations is that data from these studies could not be integrated into a single analysis. As a result, the power of evidence of these studies seems weak due to the limited data, and no convincing results can be obtained. We found varied results on the association between fracture risk and the use of anti-diabetic drugs by assessing direct comparisons, indirect comparisons, inconsistency, and heterogeneity. Unfortunately, the results of trelagliptin and voglibose were obtained based on one RCT with one fracture event. However, in this study, a comprehensive analysis was performed to detect the association between fracture risk and the use of anti-diabetic drugs by integrating data from 221,364 participants treated with nine types of anti-diabetic drugs. Compared with previous meta-analyses, the Bayesian model adopted in this study could obtain more accurate statistical results because it describes indirect comparisons between trials (25). Therefore, trelagliptin and voglibose should not be excluded. Trelagliptin raised the risk of fracture, whereas voglibose and albiglutide showed benefits with statistically significant differences. In terms of the risk of inducing fracture, voglibose (0.01%) may be the safest option, and trelagliptin (13.64%) may be the worst. RCTs with varied durations, age distributions, and sex distributions were included, but fracture risk was independent of age and sex distributions and the duration of exposure to anti-diabetic drugs.

### Glucagon-Like Peptide-1 Receptor Agonists and Fracture

In terms of fracture risk, GLP-1 receptor agonists showed benefits. A few researchers raised the doubts that GLP-1 receptor agonists may have effects on fracture risk. GLP-1 increased bone density by inhibiting bone resorption and promoting bone formation (26). But the research (included trials >12 weeks) did not support an association between the use of GLP-1 receptor agonists and the risk of fracture (27). The latest Bayesian network meta-analysis (included trials >26 weeks) suggested that exenatide showed benefits (28). Notably, according to bone biology, it is not likely that any intervention of less than 52 weeks will affect fracture risk, and therefore only RCTs with a duration of at least 52 weeks were considered in our analyses. Most of these results could not provide powerful evidence. Although GLP-1 receptor agonists did show benefits in animal models, researchers could not draw any conclusion confidently due to the limited clinical data (29, 30).

### Dipeptidyl Peptidase-4 Inhibitors and Fracture Risk

In terms of fracture risk, DPP-4 inhibitors showed varied results, and trelagliptin raised the risk of fracture with a statistical significance. A previous meta-analysis supported that DPP-4 inhibitors have neutral effects on fracture risk (27). An update meta-analysis suggested that DPP-4 inhibitors do modify the risk of fracture (31). Long-term treatment with DPP-4 inhibitors does not increase or decrease the risk of fracture (32). These findings agree with those of our Bayesian meta-analysis. Unfortunately, although our results showed that trelagliptin increased the risk of fracture, this could not be supported by available evidence. More clinical trials are needed to clarify the effect of trelagliptin on fracture events.

### Sodium–Glucose Co-Transporter 2 Inhibitors and Fracture Risk

SGLT2 inhibitors did not modify the risk of fracture with statistically significant differences. A systematic review suggested that canagliflozin is linked to an increased fracture rate (33), a conclusion that is similar with our results. One study suggested that SGLT2 inhibitors have neutral effects on fracture risk (34). In a clinical trial, SGLT2 inhibitors exhibited better benefits than other anti-diabetic drugs in T2DM patients suffering from chronic kidney disease (10). Therefore, SGLT2 inhibitors could be considered in anti-diabetic strategies for patients susceptible to fracture.

### Thiazolidinediones and Fracture Risk

Our results suggested that pioglitazone and rosiglitazone raised the risk of fracture, but no statistically significant difference was observed. Many studies showed that rosiglitazone and pioglitazone increased the risk of bone fractures (35–37). One study suggested that pioglitazone treatment does not increase the risk of fractures (38). But these studies could not provide powerful evidences due to the limited data. Therefore, thiazolidinediones should be considered carefully in patients susceptible to fracture.

### Sulfonylureas and Fracture Risk

For sulfonylureas, our results showed that that glipizide, gliclazide, glibenclamide, and glimepiride decreased the risk of fracture. One study suggested that sulfonylureas could increase the risk of fractures in the old patients with T2DM (39). Many studies have indicated that sulfonylureas have neutral effects on bone metabolism and BMD, and that they increase the amount of falling events due to the high risk of hypoglycemic episodes (40). The few available preclinical and clinical data indicate that sulfonylureas do not have detrimental effects on the bone (41). Therefore, sulfonylureas could be considered in the development of anti-diabetic strategies.

### Other Anti-Diabetic Drug and Fracture Risk

Among other anti-diabetic drugs evaluated, metformin, voglibose, and insulin showed benefits, whereas nateglinide raised the risk of fracture. Several recent studies have indicated that metformin is associated with a reduced risk of fracture (36, 42, 43), while previous studies have reported an increased risk of falling among patients using insulin (12). Therefore, metformin could be considered in patients susceptible to fracture. Nevertheless, more clinical trials are needed to clarify the effects of voglibose, insulin, and nateglinide on fracture events.

### Limitations

The following limitations of this Bayesian model should be considered. Firstly, voglibose might not be suitable for all T2DM patients due to individual differences; the probability ranking of treatments should be taken into account in selecting suitable medications. Secondly, a random-effects model was used to reduce the influence of the constraint on common variances, but this method increases the possibility of introducing biases due to heterogeneity in the included RCTs (such as doses and plasma glucose). Thirdly, a significant difference in inconsistency was noted in some direct or indirect comparisons. Inconsistency could be generated by the data available from the existing clinical trials that suffer from methodological limitations including insufficient primary endpoints and fracture events (44). Fourthly, the effects of some anti-diabetic drugs, such as licogliflozin, chlorpropamide, bromocriptine-QR, tolbutamide, and acarbose on the fracture risks, could not be evaluated due to the limited data from clinical trials. Finally, some RCTs could not be retrieved due to database or language restrictions.

## Conclusions

This comprehensive review and analysis might be helpful for researchers in investigating the relative risk of fracture related to the use of anti-diabetic drugs in future research. Further clinical trials on the association between bone fracture events and the use of anti-diabetic drugs are important since fragility fracture can seriously affect patients with diabetes. Unfortunately, the possible mechanisms of trelagliptin, voglibose, and albiglutide in promoting bone formation or inhibiting bone absorption in T2DM patients are still unclear, and there is still a lack of clinical studies to demonstrate the efficacy of trelagliptin, voglibose, and albiglutide in patients with T2DM-related fractures. Overall, we observed varied results on the association between the use of anti-diabetic drugs and fracture risk. Trelagliptin raised the risk of fracture, whereas voglibose and albiglutide showed benefits with statistically significant differences. Some anti-diabetic drugs (omarigliptin, sitagliptin, vildagliptin, saxagliptin, empagliflozin, ertugliflozin, rosiglitazone, pioglitazone, and nateglinide) may increase the risk of fracture; while others (dulaglutide, exenatide, liraglutide, semaglutide, lixisenatide, linagliptin, alogliptin, canagliflozin, dapagliflozin, glipizide, gliclazide, glibenclamide, glimepiride, metformin, and insulin) may show benefits. Many preclinical studies considered that various anti-diabetic drugs may have either aggravating or repairing effects on bone quality. Therefore, when developing T2DM treatment strategies, the clinical efficacy of various anti-diabetic drugs must also be weighed against their benefits and risks brought about by the individual differences of patients.

## Data Availability Statement

The original contributions presented in the study are included in the article/**Supplementary Material**. Further inquiries can be directed to the corresponding authors.

## Author Contributions

Y-SZ, Y-DZ, and YY contributed equally. Conceptualization: Y-SZ, B-CX, and Y-DZ. Data extraction: YY, Y-DZ, and B-CX. Formal analysis: Y-SZ and S-CC. Funding acquisition: B-CX. Investigation: Y-SZ, B-CX, and YY. Methodology: Y-SZ and YY. Project administration: Y-SZ. Resources: Y-SZ, Y-DZ, and YY. Software: B-CX and Y-DZ. Supervision: B-CX, S-CC, and Y-DZ. Validation: Y-DZ and S-CC. Writing—original draft: Y-SZ, Y-DZ, and YY. Writing—review and editing: Y-SZ, YY, and B-CX. All authors contributed to the article and approved the submitted version.

## Funding

The present study was supported by grants from the National Natural Science Foundation of China (82000842); Guangdong Basic and Applied Basic Research Foundation (2021A1515010151); Project of Administration of Traditional Chinese Medicine of Guangdong Province of China (20211403); Medical Scientific Research Foundation of Guangdong Province of China (B2020086); and Dongguan Science and Technology of Social Development Program (202050715001197).

## Conflict of Interest

The authors declare that the research was conducted in the absence of any commercial or financial relationships that could be construed as a potential conflict of interest.

## Publisher’s Note

All claims expressed in this article are solely those of the authors and do not necessarily represent those of their affiliated organizations, or those of the publisher, the editors and the reviewers. Any product that may be evaluated in this article, or claim that may be made by its manufacturer, is not guaranteed or endorsed by the publisher.
