# Supplementary material for: Effects of Anti-Diabetic Drugs on Fracture Risk: A Systematic Review and Network Meta-Analysis
Source: Front Endocrinol (Lausanne). 2021 Oct 14;12:735824. doi: 10.3389/fendo.2021.735824 (PMC8553257; doi:10.3389/fendo.2021.735824)
Supplement: Supplementary file 1 [file Table_1.docx]

| Supplemental table 1. The characteristics of included studies. | | | | | | | | | | |
| --- | --- | --- | --- | --- | --- | --- | --- | --- | --- | --- |
| Study | drug | comparator | Follow-up(Month) | Age  (years) | Masking | HbA1c(%) | N. of patients | | Fracture | |
|  |  |  |  |  |  |  | drug | Control | drug | Control |
| Matthews 2019 | vildagliptin | placebo | 60 | 54.6 | Quadruple | 6.7 (0.46) | 998 | 1001 | 4 | 12 |
| Nauck 2011 | Dapagliflozin | Glipizide | 12 | 58.6 | Quadruple | 7.72(0.870) | 406 | 408 | 1 | 4 |
| Mathieu 2015 | dapagliflozin | Placebo | 12 | 55.0 | Triple | 8.20 (0.97) | 160 | 160 | 0 | 1 |
| NCT02906709 | Omarigliptin | Placebo | 12 | 60.9 | Double | 8.82 (0.71) | 123 | 61 | 1 | 0 |
| Suzuki 2017 | dulaglutide | Liraglutide | 12 | 57.91 | Quadruple | 8.2 (0.8) | 280 | 137 | 3 | 2 |
| Hollander 2017 | Ertugliflozin | Glimepiride | 24 | 57.8 | Double | 7.79 (0.60) | 880 | 435 | 1 | 1 |
| Li 2019 | Dulaglutide | Insulin | 12 | 55.44 | Open Label | 8.33(1.08) | 515 | 253 | 6 | 2 |
| Gantz 2017 | Omarigliptin | Placebo | 54 | 63.6 | Triple | 8.01 (0.87) | 2092 | 2100 | 20 | 13 |
| Home 2017 | Omarigliptin | Placebo | 13 | 57.0 | Triple | 8.1 (1.0) | 165 | 164 | 0 | 2 |
| Dagogo-Jack 2014 | Ertugliflozin | Placebo | 12 | 58.3 | Open Label | 8.03 (0.88) | 309 | 153 | 2 | 0 |
| Sheu 2015 | Omarigliptin | Placebo | 20 | 55.9 | Triple | 8.1 (0.9) | 1089 | 80 | 0 | 1 |
| Shankar 2017 | Omarigliptin | Glimepiride | 24 | 56.8 | Triple | 8.04 (0.88) | 201 | 201 | 1 | 0 |
| NCT02512068 | Trelagliptin | Placebo | 12 | 65.8 | Quadruple | NA | 55 | 48 | 1 | 0 |
| Nowicki 2011 | Saxagliptin | Placebo | 12 | 66.2 | Triple | 8.5(1.2) | 85 | 85 | 1 | 0 |
| Gordon 2016 | Alogliptin | Glipizide | 24 | 55.4 | Double | 7.60(0.607) | 1751 | 869 | 6 | 4 |
| Gallwitz 2012 | exenatide | Glimepiride | 54 | 56.8 | Open Label | 7.4 (0.70) | 511 | 508 | 6 | 5 |
| Henry 2013 | Sitagliptin | Pioglitazone | 12 | 50.3 | Double | 8.8 (1.1) | 231 | 693 | 1 | 1 |
| Goldstein 2008 | Sitagliptin | Metformin | 24 | 53.2 | Double | 9.0 (1.2) | 179 | 364 | 0 | 3 |
| Mosenzon 2015 | Saxagliptin | Placebo | 35 | 65.0 | Quadruple | 7.7(0.6) | 8280 | 8212 | 101 | 86 |
| Dobs 2013 | Sitagliptin | Placebo | 12 | 54.8 | Double | 8.8 (1.0) | 170 | 92 | 0 | 1 |
| NCT00367055 | Rosiglitazone | Gliclazide | 36 | 58.1 | Open Label | 7.4 (0.57) | 43 | 41 | 0 | 1 |
| Schernthaner 2015 | Saxagliptin | Glimepiride | 12 | 72.7 | Double | 7.60 (0.66) | 359 | 359 | 4 | 1 |
| Tuttle 2018 | Dulaglutide | Insulin | 12 | 64.7 | Open Label | 8.57(0.973) | 382 | 194 | 0 | 3 |
| Terauchi 2017 | Sitagliptin | Glimepiride | 12 | 70.8 | Open Label | 7.48 (0.67) | 148 | 143 | 1 | 0 |
| NCT00374907 | Saxagliptin | Placebo | 24 | 55 | Double | NA | 20 | 16 | 1 | 0 |
| Pinget 2013 | Lixisenatide | Placebo | 24 | 56.0 | Double | 8.07 (0.86) | 323 | 161 | 2 | 0 |
| Barnett 2013 | Saxagliptin | Placebo | 12 | 57.3 | Double | 8.7 (0.9) | 304 | 151 | 1 | 0 |
| Göke 2013 | Saxagliptin | Glipizide | 12 | 57.59 | Quadruple | 7.46(0.045) | 428 | 430 | 4 | 2 |
| Bethel 2017 | Sitagliptin | Placebo | 68 | 74.9 | Double | 7.2 (0.5) | 7266 | 7274 | 58 | 59 |
| Weinstock 2015 | Dulaglutide | Sitagliptin | 24 | 53.75 | Quadruple | 8.13 (1.09) | 710 | 315 | 4 | 2 |
| Jaiswal 2015 | Exenatide | Insulin | 12 | 54 | Open Label | 8.2(1.1) | 22 | 24 | 1 | 1 |
| Hernandez 2018 | Albiglutide | Placebo | 32 | 64.2 | Quadruple | 8.7 (1.5) | 4717 | 4715 | 30 | 31 |
| Bilezikian 2013 | Rosiglitazone | Metformin | 12 | 64.0 | Double | 6.8 (0.74) | 190 | 195 | 0 | 1 |
| Neuen 2018 | Canagliflozin | Placebo | 36 | 64 | Quadruple | 8.1 ( 1.3) | 2904 | 2903 | 38 | 35 |
| Rodbard 2019 | Semaglutide | Empagliflozin | 12 | 58 | Open Label | 8.1 (0.9) | 410 | 409 | 0 | 2 |
| Husain 2019 | Semaglutide | placebo | 18 | 66 | Double | 8.2(1.6) | 1591 | 1591 | 12 | 15 |
| Lingvay 2019 | Semaglutide | Canagliflozin | 12 | 57.5 | Double | 8.3 (1.0) | 392 | 394 | 1 | 2 |
| Pratley 2019 | semaglutide | Liraglutide | 12 | 56 | Double | 8.0 (0.7) | 285 | 284 | 1 | 2 |
| Umpierrez 2014 | Dulaglutide | Metformin | 12 | 55.26 | Quadruple | 7.60 (0.87) | 539 | 268 | 1 | 0 |
| NCT02589639 | Empagliflozin | Placebo | 12 | 58 | Open Label | 8.8 (0.7) | 176 | 90 | 1 | 3 |
| Weissman 2014 | Albiglutide | insulin | 36 | 54.7 | Open Label | 8.31(0.92) | 504 | 241 | 3 | 0 |
| Boustani 2018 | Dulaglutide | insulin | 12 | 59.9 | Open Label | 8.46 (1.05) | 588 | 296 | 3 | 3 |
| Home 2017 | Albiglutide | Placebo | 36 | 53.1 | Double | 8.1 (0.9) | 200 | 101 | 1 | 0 |
| Yabe 2020 | Semaglutide | Dulaglutide | 12 | 61 | Open Label | 8.3(0.9) | 393 | 65 | 1 | 0 |
| Kawamori 2018 | Empagliflozin | Placebo | 12 | 59.8 | Double | 8.3 (0.74) | 182 | 93 | 2 | 0 |
| Boustani 2016 | Dulaglutide | Exenatide | 12 | 55.45 | Quadruple | 8.07 (1.31) | 599 | 278 | 0 | 1 |
| Giorgino 2015 | Dulaglutide | Insulin | 18 | 57.21 | Open Label | 8.14 (0.99) | 545 | 262 | 1 | 0 |
| Home 2017 | Sitagliptin | Placebo | 36 | 56.1 | Double | 8.3 (0.9) | 302 | 101 | 2 | 0 |
| Home 2017 | Albiglutide | Glimepiride | 36 | 54.4 | Double | 8.3 (0.9) | 302 | 307 | 0 | 2 |
| Watts 2015 | Canagliflozin | Placebo | 12 | 68.2 | Quadruple | 8.0 (0.9) | 179 | 90 | 0 | 2 |
| NCT03235050 | Liraglutide | Placebo | 12 | 57.3 | Triple | 8.2 (1.2) | 110 | 112 | 0 | 1 |
| Pieber 2019 | semaglutide | sitagliptin | 24 | 58 | Open Label | 8.3 (0.6) | 606 | 347 | 4 | 0 |
| Kaku 2019 | liraglutide | insulin | 12 | 57.8 | Open Label | NA | 273 | 271 | 1 | 0 |
| Home 2017 | Albiglutide | Pioglitazone | 36 | 55.7 | Double | 8.2 (0.9) | 271 | 277 | 3 | 2 |
| Ahrén 2017 | semaglutide | sitagliptin | 12 | 54.6 | Double | 8.0(0.9) | 816 | 407 | 0 | 1 |
| Ridderstråle 2015 | empagliflozin | Glimepiride | 48 | 55.7 | Double | 7.92 (0.81) | 765 | 780 | 14 | 4 |
| Jódar 2020 | semaglutide | placebo | 24 | 64.6 | Double | 8.7( 1.5) | 1648 | 1649 | 9 | 12 |
| Zinman 2014 | Empagliflozin | Placebo | 60 | 63.2 | Double | 8.1 (0.8) | 4687 | 2333 | 58 | 37 |
| Ahmann 2017 | Semaglutide | exenatide | 12 | 56.7 | Open Label | 8.3 (0.95) | 404 | 405 | 1 | 0 |
| Young 2014 | albiglutide | sitagliptin | 12 | 63.5 | Double | NA | 249 | 246 | 2 | 0 |
| Nauck 2007 | Sitagliptin | glipizide | 24 | 56.6 | Double | 7.7 (0.9) | 588 | 584 | 3 | 3 |
| Bolli 2013 | Lixisenatide | Placebo | 25 | 57.5 | Double | 8.04（0.86） | 644 | 320 | 6 | 4 |
| Riddle 2013 | Lixisenatide | Placebo | 30 | 56.9 | Quadruple | 8.40（0.87） | 328 | 167 | 2 | 1 |
| Rosenstock 2014 | Lixisenatide | Placebo | 28 | 57.8 | Double | 8.25 (0.85) | 574 | 285 | 4 | 1 |
| Nauck 2006 | exenatide | insulin | 12 | 58.5 | Open Label | 8.59 (0.07) | 253 | 248 | 1 | 0 |
| Heine 2005 | Exenatide | insulin | 12 | 57.96 | Open Label | 8.13 (0.07) | 282 | 267 | 0 | 1 |
| DeFronzo 2018 | Empagliflozin | Linagliptin | 12 | 55.0 | Double | 8.01 (0.90) | 551 | 267 | 2 | 0 |
| Rosenstock 2018 | Linagliptin | Placebo | 52 | 65.6 | Double | 7.9 (1.0) | 3494 | 3485 | 50 | 43 |
| Rosenstock 2014 | empagliflozin | Placebo | 12 | 55.3 | Double | 8.39 ( 0.05) | 375 | 188 | 0 | 1 |
| Rosenstock 2019 | Linagliptin | Glimepiride | 102 | 64.2 | Double | 7.2 (0.6) | 3023 | 3010 | 79 | 92 |
| Gough 2014 | liraglutide | insulin | 12 | 54.9 | Open Label | 8.3 (0.9) | 412 | 412 | 0 | 1 |
| Roden 2015 | Empagliflozin | Placebo | 18 | 55.5 | Double | 7.88 (0.82) | 1655 | 822 | 14 | 2 |
| Zannad 201573 | Alogliptin | Placebo | 41 | 60.7 | Quadruple | 9.16(8.159) | 2701 | 2679 | 13 | 21 |
| Barnett 2014 | empagliflozin | Placebo | 15 | 64.1 | Double | 8.03 (0.79) | 419 | 319 | 1 | 4 |
| Pratley 2010 | liraglutide | sitagliptin | 18 | 55.0 | Open Label | 8.4 (0.8) | 439 | 219 | 0 | 1 |
| Arjona 2012 | Sitagliptin | Glipizide | 12 | 64.2 | Triple | 7.8 ( 0.7 ) | 212 | 210 | 1 | 1 |
| Arjona 2013 | Sitagliptin | Glipizide | 12 | 58.5 | Double | 7.89 (0.74) | 64 | 65 | 2 | 0 |
| Davies 2015 | Liraglutide | placebo | 15 | 54.7 | Double | 7.9 (0.8) | 632 | 212 | 1 | 0 |
| Ratner 2008 | Glipizide | rosiglitazone | 18 | 61.8 | Triple | 7.2 (0.87) | 337 | 331 | 1 | 1 |
| NCT01011868 | Empagliflozin | Placebo | 18 | 58.1 | Double | NA | 324 | 170 | 1 | 1 |
| Yki-Järvinen 2013 | Linagliptin | Placebo | 12 | 60.4 | Double | 8.30 (0.85) | 631 | 630 | 5 | 5 |
| Gallwitz 2012 | Linagliptin | Glimepiride | 24 | 59.8 | Double | 7.69 (0.87) | 776 | 775 | 9 | 4 |
| Leiter 2014 | Canagliflozin | Glimepiride | 24 | 56.3 | Quadruple | 7.8 (0.8) | 968 | 482 | 9 | 2 |
| Schernthaner 2013 | Canagliflozin | Sitagliptin | 12 | 56.6 | Triple | 8.1(0.9) | 377 | 378 | 2 | 1 |
| Lavalle 2013 | Canagliflozin | Sitagliptin | 12 | 55.5 | Triple | 7.9 ( 0.9) | 735 | 366 | 1 | 0 |
| Jones 2015 | Rosiglitazone | Metformin | 90 | 57.2 | Open Label | 9.5 (2.1) | 1103 | 1122 | 29 | 22 |
| Jones 2015 | Rosiglitazone | Metformin | 90 | 59.7 | Open Label | 10.1 (2.3) | 1103 | 1122 | 31 | 20 |
| NCT02564926 | Dapagliflozin | Glimepiride | 12 | 55.7 | Open Label | NA | 60 | 61 | 2 | 1 |
| Neal 2013 | Canagliflozin | Placebo | 96 | 62.3 | Quadruple | 8.2 (0.9) | 2886 | 1441 | 16 | 101 |
| Bethel 2009 | Nateglinide | Placebo | 72 | 63.7 | Double | 7.8 (0.7) | 2297 | 2283 | 57 | 50 |
| Bethel 2009 | Nateglinide | Placebo | 72 | 63.9 | Double | NA | 2305 | 2316 | 67 | 42 |
| Araki 2019 | Linagliptin | Placebo | 12 | 72.5 | Double | 8.2 (0.8) | 151 | 151 | 0 | 2 |
| Gerstein 2019 | Dulaglutide | Placebo | 84 | 66.2 | Double | 7.3 (1.1) | 4943 | 4949 | 149 | 158 |
| NCT01204294 | Linagliptin | Metformin | 12 | 59.3 | Open Label | 8.12 (0.78) | 450 | 124 | 3 | 0 |
| Sullivan 2009 | liraglutide | glimepiride | 24 | 53.4 | Double | 8.3 (1.1) | 497 | 248 | 2 | 1 |
| Rosenstock 2014 | Albiglutide | Insulin | 12 | 56.3 | Open Label | 8.5 (0.9) | 285 | 281 | 0 | 3 |
| Johansen 2012 | Linagliptin | voglibose | 12 | 58.5 | Double | 8.0 (0.9) | 319 | 162 | 1 | 0 |
| Nauck 2008 | liraglutide | glimepiride | 24 | 57.3 | Double | 8.4 (0.9) | 725 | 244 | 3 | 1 |
| Jardine 2017 | Canagliflozin | Placebo | 54 | 63. | Double | 8.3 (1.3) | 2200 | 2197 | 31 | 22 |
| Bentley 2015 | Lixisenatide | Placebo | 52 | 60.6 | Triple | 7.68 (1.30) | 3031 | 3032 | 16 | 28 |
| Ferrannini 2013 | Empagliflozin | Metformin | 18 | 56.8 | Open Label | 8.0 (0.87) | 215 | 56 | 2 | 1 |
| Leiter 2016 | Dapagliflozin | Placebo | 62 | 64.0 | Quadruple | 8.1(0.8) | 8574 | 8569 | 190 | 196 |
| Grey 2014 | Pioglitazone | Placebo | 12 | 64 | Double | 7.34 (0.32) | 43 | 43 | 2 | 0 |
| Bray 2013 | Pioglitazone | Placebo | 33.6 | 53 | Double | 5.0 (0.40) | 303 | 299 | 9 | 8 |
| Bone 2013 | Pioglitazone | Placebo | 12 | 59 | Double | NA | 78 | 78 | 1 | 3 |
| Borges 2011 | Rosiglitazone | Placebo | 19 | 52.5 | Double | 8.6 (0.9) | 344 | 334 | 5 | 5 |
| Home 2009 | Rosiglitazone | metformin | 66 | 59.8 | Double | 8.0 (0.7) | 2220 | 2227 | 185 | 118 |
| Tloman K 2009 | Pioglitazone | Glibenclamide | 36 | 54 | Quadruple | 9.5 (2.0) | 1051 | 1046 | 30 | 27 |
| Kahn 2008 | Pioglitazone | glimepiride | 48 | 56.3 | Double | 7.36 (0.93) | 270 | 273 | 8 | 0 |
| Seufert1 2008 | Pioglitazone | gliclazide | 24 | 56 | Double | 8.63 (0.88) | 317 | 313 | 1 | 1 |
| Seufert2 2008 | Pioglitazone | metformin | 24 | 60 | Double | 8.81 (0.98) | 319 | 320 | 0 | 2 |
| Dormandy 2005 | Pioglitazone | Placebo | 34.5 | 61.9 | Double | 9.2 (1.26) | 2605 | 2633 | 74 | 60 |
| NCT00707993 | Alogliptin | Glipizide | 12 | 69.8 | Quadruple | 7.9 (1.85) | 222 | 219 | 2 | 1 |
| Jabbour 2018 | Exentide | Dapagliflozin | 12 | 54.5 | Double | 9.3 (1.0) | 230 | 233 | 1 | 1 |
| McGill 2014 | Linagliptin | placebo | 12 | 64.0 | Double | 8.2 (1.0) | 68 | 65 | 2 | 0 |
| Tack 2019 | liraglutide | placebo | 42 | 64.2 | Double | 8.7 (1.5) | 4668 | 4672 | 31 | 55 |
| Rosenstock 2018 | Empagliflozin | Placebo | 12 | 44.2 | Double | 8.13 ( 0.57) | 487 | 243 | 2 | 1 |

| **Supplemental table 2** - Qualitative analysis through Jadad Score. | | | | | | |
| --- | --- | --- | --- | --- | --- | --- |
|  | Randomisation | Method of randomisation | Double blinding | Description of withdrawals and dropouts | Total score | Quality |
| Matthews 2019 | 2 | 2 | 2 | 1 | 7 | High |
| Nauck 2011 | 2 | 2 | 2 | 1 | 7 | High |
| Mathieu 2015 | 2 | 2 | 2 | 1 | 7 | High |
| NCT02906709 | 2 | 2 | 2 | 1 | 7 | High |
| Suzuki 2017 | 2 | 2 | 2 | 1 | 7 | High |
| Hollander 2017 | 2 | 2 | 2 | 1 | 7 | High |
| Li 2019 | 2 | 2 | 0 | 1 | 5 | High |
| Gantz 2017 | 2 | 2 | 2 | 1 | 7 | High |
| Home 2017 | 2 | 2 | 2 | 1 | 7 | High |
| Dagogo-Jack 2014 | 2 | 2 | 0 | 1 | 5 | High |
| Sheu 2015 | 2 | 2 | 2 | 1 | 7 | High |
| Shankar 2017 | 2 | 2 | 2 | 1 | 7 | High |
| NCT02512068 | 2 | 2 | 2 | 1 | 7 | High |
| Nowicki 2011 | 2 | 2 | 2 | 1 | 7 | High |
| Gordon 2016 | 2 | 2 | 2 | 1 | 7 | High |
| Gallwitz 2012 | 2 | 2 | 0 | 1 | 5 | High |
| Henry 2013 | 2 | 2 | 2 | 1 | 7 | High |
| Goldstein 2008 | 2 | 2 | 2 | 1 | 7 | High |
| Mosenzon 2015 | 2 | 2 | 2 | 1 | 7 | High |
| Dobs 2013 | 2 | 2 | 2 | 1 | 7 | High |
| NCT00367055 | 2 | 2 | 0 | 1 | 5 | High |
| Schernthaner 2015 | 2 | 2 | 2 | 1 | 7 | High |
| Tuttle 2018 | 2 | 2 | 0 | 1 | 5 | High |
| Terauchi 2017 | 2 | 2 | 0 | 1 | 5 | High |
| NCT00374907 | 2 | 2 | 2 | 1 | 7 | High |
| Pinget 2013 | 2 | 2 | 2 | 1 | 7 | High |
| Barnett 2013 | 2 | 2 | 2 | 1 | 7 | High |
| Göke 2013 | 2 | 2 | 2 | 1 | 7 | High |
| Bethel 2017 | 2 | 2 | 2 | 1 | 7 | High |
| Weinstock 2015 | 2 | 2 | 2 | 1 | 7 | High |
| Jaiswal 2015 | 2 | 2 | 0 | 1 | 7 | High |
| Hernandez 2018 | 2 | 2 | 2 | 1 | 7 | High |
| Bilezikian 2013 | 2 | 2 | 2 | 1 | 7 | High |
| Neuen 2018 | 2 | 2 | 2 | 1 | 7 | High |
| Rodbard 2019 | 2 | 2 | 0 | 1 | 5 | High |
| Husain 2019 | 2 | 2 | 2 | 1 | 7 | High |
| Lingvay 2019 | 2 | 2 | 2 | 1 | 7 | High |
| Pratley 2019 | 2 | 2 | 2 | 1 | 7 | High |
| Umpierrez 2014 | 2 | 2 | 2 | 1 | 7 | High |
| NCT02589639 | 2 | 2 | 0 | 1 | 5 | High |
| Weissman 2014 | 2 | 2 | 0 | 1 | 5 | High |
| Boustani 2018 | 2 | 2 | 0 | 1 | 5 | High |
| Home 2017 | 2 | 2 | 2 | 1 | 7 | High |
| Yabe 2020 | 2 | 2 | 0 | 1 | 5 | High |
| Kawamori 2018 | 2 | 2 | 2 | 1 | 7 | High |
| Boustani 2016 | 2 | 2 | 2 | 1 | 7 | High |
| Giorgino 2015 | 2 | 2 | 0 | 1 | 5 | High |
| Home 2017 | 2 | 2 | 2 | 1 | 7 | High |
| Home 2017 | 2 | 2 | 2 | 1 | 7 | High |
| Watts 2015 | 2 | 2 | 2 | 1 | 7 | High |
| NCT03235050 | 2 | 2 | 2 | 1 | 7 | High |
| Pieber 2019 | 2 | 2 | 0 | 1 | 5 | High |
| Kaku 2019 | 2 | 2 | 0 | 1 | 5 | High |
| Home 2017 | 2 | 2 | 2 | 1 | 7 | High |
| Ahrén 2017 | 2 | 2 | 2 | 1 | 7 | High |
| Ridderstråle 2015 | 2 | 2 | 2 | 1 | 7 | High |
| Jódar 2020 | 2 | 2 | 2 | 1 | 7 | High |
| Zinman 2014 | 2 | 2 | 2 | 1 | 7 | High |
| Ahmann 2017 | 2 | 2 | 0 | 1 | 5 | High |
| Young 2014 | 2 | 2 | 2 | 1 | 7 | High |
| Nauck 2007 | 2 | 2 | 2 | 1 | 7 | High |
| Bolli 2013 | 2 | 2 | 2 | 1 | 7 | High |
| Riddle 2013 | 2 | 2 | 2 | 1 | 7 | High |
| Rosenstock 2014 | 2 | 2 | 2 | 1 | 7 | High |
| Nauck 2006 | 2 | 2 | 0 | 1 | 5 | High |
| Heine 2005 | 2 | 2 | 0 | 1 | 5 | High |
| DeFronzo 2018 | 2 | 2 | 2 | 1 | 7 | High |
| Rosenstock 2018 | 2 | 2 | 2 | 1 | 7 | High |
| Rosenstock 2014 | 2 | 2 | 2 | 1 | 7 | High |
| Rosenstock 2019 | 2 | 2 | 2 | 1 | 7 | High |
| Gough 2014 | 2 | 2 | 0 | 1 | 5 | High |
| Roden 2015 | 2 | 2 | 2 | 1 | 7 | High |
| Zannad 2015 | 2 | 2 | 2 | 1 | 7 | High |
| Barnett 2014 | 2 | 2 | 2 | 1 | 7 | High |
| Pratley 2010 | 2 | 2 | 0 | 1 | 5 | High |
| Arjona 2012 | 2 | 2 | 2 | 1 | 7 | High |
| Arjona 2013 | 2 | 2 | 2 | 1 | 7 | High |
| Davies 2015 | 2 | 2 | 2 | 1 | 7 | High |
| Ratner 2008 | 2 | 2 | 2 | 1 | 7 | High |
| NCT01011868 | 2 | 2 | 2 | 1 | 7 | High |
| Yki-Järvinen 2013 | 2 | 2 | 2 | 1 | 7 | High |
| Gallwitz 2012 | 2 | 2 | 2 | 1 | 7 | High |
| Leiter 2014 | 2 | 2 | 2 | 1 | 7 | High |
| Schernthaner 2013 | 2 | 2 | 2 | 1 | 7 | High |
| Lavalle 2013 | 2 | 2 | 2 | 1 | 7 | High |
| Jones 2015 | 2 | 2 | 0 | 1 | 5 | High |
| Jones 2015 | 2 | 2 | 0 | 1 | 5 | High |
| NCT02564926 | 2 | 2 | 0 | 1 | 5 | High |
| Neal 2013 | 2 | 2 | 2 | 1 | 7 | High |
| Bethel 2009 | 2 | 2 | 2 | 1 | 7 | High |
| Bethel 2009 | 2 | 2 | 2 | 1 | 7 | High |
| Araki 2019 | 2 | 2 | 2 | 1 | 7 | High |
| Gerstein 2019 | 2 | 2 | 2 | 1 | 7 | High |
| NCT01204294 | 2 | 2 | 0 | 1 | 5 | High |
| Sullivan 2009 | 2 | 2 | 2 | 1 | 7 | High |
| Rosenstock 2014 | 2 | 2 | 0 | 1 | 5 | High |
| Johansen 2012 | 2 | 2 | 2 | 1 | 7 | High |
| Nauck 2008 | 2 | 2 | 2 | 1 | 7 | High |
| Jardine 2017 | 2 | 2 | 2 | 1 | 7 | High |
| Bentley 2015 | 2 | 2 | 2 | 1 | 7 | High |
| Ferrannini 2013 | 2 | 2 | 0 | 1 | 5 | High |
| Leiter 2016 | 2 | 2 | 2 | 1 | 7 | High |
| NCT00707993 | 2 | 2 | 2 | 1 | 7 | High |
| Jabbour 2018 | 2 | 2 | 2 | 1 | 7 | High |
| McGill 2014 | 2 | 2 | 2 | 1 | 7 | High |
| Tack 2019 | 2 | 2 | 2 | 1 | 7 | High |
| Grey 2014 | 2 | 2 | 2 | 1 | 7 | High |
| Bray 2013 | 2 | 2 | 2 | 1 | 7 | High |
| Bone 2013 | 2 | 2 | 2 | 1 | 7 | High |
| Borges 2011 | 2 | 2 | 2 | 1 | 7 | High |
| Home 2009 | 2 | 2 | 2 | 1 | 7 | High |
| Tloman K 2009 | 2 | 2 | 2 | 1 | 7 | High |
| Kahn 2008 | 2 | 2 | 2 | 1 | 7 | High |
| Seufert1 2008 | 2 | 2 | 2 | 1 | 7 | High |
| Seufert2 2008 | 2 | 2 | 2 | 1 | 7 | High |
| Dormandy 2005 | 2 | 2 | 2 | 1 | 7 | High |
| Rosenstock 2018 | 2 | 2 | 2 | 1 | 7 | High |

| Supplemental table 3 - Probability of treatments rankings | | |
| --- | --- | --- |
| Treatment | Bone fracture events | |
|  | Fracture probabilities | Rank |
| voglibose | 0.01% | 1 |
| albiglutide | 0.23% | 2 |
| glimepiride | 0.33% | 3 |
| canagliflozin | 1.04% | 4 |
| semaglutide | 1.36% | 5 |
| glipizide | 1.36% | 6 |
| insulin | 1.68% | 7 |
| liraglutide | 2.30% | 8 |
| gliclazide | 3.11% | 9 |
| alogliptin | 3.19% | 10 |
| metformin | 3.21% | 11 |
| dapagliflozin | 3.28% | 12 |
| linagliptin | 3.34% | 13 |
| lixisenatide | 3.46% | 14 |
| dulaglutide | 3.82% | 15 |
| exenatide | 3.95% | 16 |
| glibenclamide | 3.99% | 17 |
| pioglitazone | 4.06% | 18 |
| vildagliptin | 4.25% | 19 |
| empagliflozin | 4.41% | 20 |
| rosiglitazone | 4.67% | 21 |
| sitagliptin | 4.75% | 22 |
| omarigliptin | 5.15% | 23 |
| nateglinide | 5.33% | 24 |
| saxagliptin | 5.59% | 25 |
| ertugliflozin | 5.73% | 26 |
| trelagliptin | 13.64% | 27 |
| Note: Ranking: probability of being the best treatment, of being the second best, the third best and so on, among the 26 treatments. | | |

Supplemental table 4 - Node split method for inconsistence check in network meta-analysis.

| Comparison | Direct | |  |  | Indirect | |  | Difference | | *P* |
| --- | --- | --- | --- | --- | --- | --- | --- | --- | --- | --- |
|  | RR | 95% CI |  |  | RR | 95% CI |  | RR | 95% CI |  |
| 10 vs. 17 | -31 | (-1.3e+02,-2.8) |  |  | -0.34 | -1.3,0.67 |  | -0.57 | -1.5,0.39 | <0.05 |
| 10 vs. 21 | -45 | -1.4e+02,-3.3 |  |  | -0.95 | -1.9,-0.072 |  | -1 | -1.9,-0.2 | <0.05 |
| 16 vs. 18 | -52 | -1.4e+02,-3.7 |  |  | -0.12 | -1.0,0.82 |  | -0.27 | -1.2,0.59 | <0.05 |
| 3 vs.21 | -40 | -1.4e+02,-2.9 |  |  | -0.85 | -2.2,0.45 |  | -1.1 | -2.3,0.15 | <0.05 |
| 5 vs. 21 | -0.19 | -3.8,2.7 |  |  | -39 | -1.3e+02,-3.1 |  | -1.7 | -5.1,0.57 | <0.05 |
| 4 vs. 23 | -33 | ( -1.5e+02,-1.2) |  |  | 0.14 | -1.2,1.5 |  | -0.1 | -1.3,1.1 | <0.05 |
| 3 vs. 27 | -0.15 | -1.3,0.99 |  |  | -45 | -1.5e+02,-2.4 |  | -0.32 | -1.4,0.76 | <0.05 |
| 5 vs. 27 | -32 | 1.2e+02,-2.1 |  |  | 0.63 | -3.2,3.6 |  | -0.91 | -4.4,1.3 | <0.05 |
| Note: 3="omarigliptin"; 4="dulaglutide"; 5="ertugliflozin"; 10="sitagliptin"; 16="empagliflozin"; 17="liraglutide"; 18="linagliptin"; 21="glimepiride"; 23="metformin"; 27="glibenclamide". | | | | | | | | | | |

| Supplemental table 5 - Heterogeneity check in network meta-analysis. | | | | | | | | |
| --- | --- | --- | --- | --- | --- | --- | --- | --- |
| Comparison | | | Direct (heterogeneity) | | Indirect (heterogeneity) | | *P* | |
| Treatment1 | | Treatment2 |  |  |  |  |  |  |
| 10 | 14 | | 0.00 | 0.00 | | 0.64 | |  |
| 10 | 15 | | 40.97 | 47.44 | | 0.84 | |  |
| 10 | 19 | | 20.72 | 0.00 | | 0.58 | |  |
| 10 | 27 | | 32.69 | 29.30 | | 0.88 | |  |
| 11 | 23 | | 0.00 | 0.00 | | 0.96 | |  |
| 12 | 27 | | 50.67 | 42.88 | | 0.90 | |  |
| 13 | 25 | | 60.77 | 48.76 | | 0.77 | |  |
| 13 | 27 | | 68.12 | 9.40 | | 0.83 | |  |
| 14 | 27 | | 55.94 | 3.68 | | 0.40 | |  |
| 15 | 27 | | 0.00 | 0.00 | | 0.65 | |  |
| 16 | 27 | | 31.68 | 36.73 | | 0.64 | |  |
| 17 | 21 | | 0.00 | 0.00 | | 0.94 | |  |
| 17 | 25 | | 57.04 | 0.00 | | 0.35 | |  |
| 17 | 27 | | 52.91 | 1.82 | | 0.86 | |  |
| 18 | 21 | | 69.27 | 93.49 | | 0.42 | |  |
| 18 | 27 | | 7.17 | 8.03 | | 0.32 | |  |
| 19 | 8 | | 6.81 | 0.00 | | 0.73 | |  |
| 2 | 27 | | 0.00 | 0.00 | | 0.90 | |  |
| 20 | 27 | | 37.31 | 37.28 | | 0.93 | |  |
| 22 | 27 | | 1.94 | 0.00 | | 0.62 | |  |
| 25 | 4 | | 38.42 | 34.82 | | 0.34 | |  |
| 25 | 9 | | 7.38 | 0.00 | | 0.38 | |  |
| 27 | 3 | | 48.39 | 28.77 | | 0.55 | |  |
| 27 | 7 | | 65.58 | 38.37 | | 0.72 | |  |
| Note:1="vildagliptin"; 2="dapagliflozin"; 3="omarigliptin"; 4="dulaglutide"; 5="ertugliflozin"; 6="trelagliptin"; 7="saxagliptin"; 8="alogliptin"; 9="exenatide"; 10="sitagliptin"; 11="rosiglitazone"; 12="lixisenatide"; 13="albiglutide "; 14="canagliflozin"; 15="semaglutide"; 16="empagliflozin"; 17="liraglutide"; 18="linagliptin"; 19="glipizide"; 20="nateglinide"; 21="glimepiride"; 22="pioglitazone"; 23="metformin"; 24="gliclazide"; 25="insulin"; 26="voglibose"; 27="glibenclamide". The global heterogeneity parameter *I*^2^ values were 44% calculated by R software. To help identify comparisons where heterogeneity is present, the heterogeneity of all direct and indirect comparisons were observed in the table. | | | | | | | |  |

| **Supplemental table 6 - Sensitivity analysis of network meta-analysis for risk of bone fracture** | | | | | | | | | | | | | | | | | | | | | | | | | | | | | |
| --- | --- | --- | --- | --- | --- | --- | --- | --- | --- | --- | --- | --- | --- | --- | --- | --- | --- | --- | --- | --- | --- | --- | --- | --- | --- | --- | --- | --- | --- |
| Treatment | albiglutide | dulaglutide | exenatide | lixisenatide | liraglutide | semaglutide | vildagliptin | omarigliptin | trelagliptin | saxagliptin | alogliptin | sitagliptin | linagliptin | dapagliflozin | ertugliflozin | canagliflozin | empagliflozin | glipizide | glimepiride | glibenclamide | gliclazide | pioglitazone | rosiglitazone | nateglinide | metformin | insulin | voglibose | placebo | |
| albiglutide | NA | 0.43(0.07, 2.51) | 0.41(0.06, 2.86) | 0.36(0.05, 2.13) | 0.53(0.08, 3.11) | 0.59(0.1, 3.41) | 0.34(0.05, 2.01) | 0.29(0.04, 2) | 0.01(0, 0.04) | 0.19(0.03, 1.12) | 0.51(0.07, 3.43) | 0.3(0.05, 1.62) | 0.43(0.07, 2.38) | 0.44(0.07, 2.67) | 0.15(0, 2.48) | 0.63(0.11, 3.4) | 0.33(0.06, 1.78) | 0.59(0.09, 3.56) | 0.86(0.15, 4.71) | 0.39(0.04, 3.33) | 0.52(0.03, 9.06) | 0.34(0.06, 1.88) | 0.33(0.05, 2.17) | 0.59(0.09, 3.56) | 0.48(0.07, 3.18) | 0.58(0.09, 3.77) | 1.22(1.1, 2.88) | 0.28(0.08, 0.96) | |
| dulaglutide | 2.31(0.4, 14.57) | NA | 0.96(0.28, 3.17) | 0.83(0.25, 2.63) | 1.23(0.46, 3.31) | 1.38(0.49, 3.9) | 0.78(0.26, 2.29) | 0.68(0.18, 2.63) | 0.02(0, 0.08) | 0.45(0.13, 1.43) | 1.18(0.31, 4.48) | 0.7(0.27, 1.78) | 1(0.36, 2.81) | 1.01(0.32, 3.27) | 0.37(0.01, 4.13) | 1.47(0.54, 3.91) | 0.77(0.28, 2.07) | 1.35(0.41, 4.46) | 2(0.78, 5.43) | 0.9(0.17, 4.76) | 1.21(0.11, 14.91) | 0.79(0.28, 2.22) | 0.75(0.22, 2.66) | 1.35(0.41, 4.46) | 1.12(0.34, 3.86) | 1.35(0.55, 3.4) | 1.81(1.36, 5.84) | 0.92(0.42, 2.07) | |
| exenatide | 2.42(0.35, 17.46) | 1.04(0.32, 3.53) | NA | 0.86(0.2, 3.47) | 1.31(0.36, 4.75) | 1.43(0.41, 5.16) | 0.82(0.21, 3.07) | 0.71(0.15, 3.38) | 0.03(0, 0.09) | 0.46(0.11, 1.84) | 1.24(0.26, 5.72) | 0.73(0.21, 2.54) | 1.05(0.3, 3.62) | 1.06(0.29, 3.94) | 0.38(0.01, 4.68) | 1.53(0.43, 5.36) | 0.8(0.23, 2.78) | 1.41(0.35, 5.89) | 2.08(0.68, 6.52) | 0.94(0.16, 5.74) | 1.26(0.11, 16.99) | 0.82(0.23, 2.96) | 0.79(0.18, 3.53) | 1.41(0.35, 5.89) | 1.17(0.27, 5.18) | 1.4(0.44, 4.74) | 2.25(1.54, 6.95) | 0.97(0.31, 2.97) | |
| lixisenatide | 2.79(0.47, 18.22) | 1.21(0.38, 4.05) | 1.17(0.29, 4.88) | NA | 1.49(0.48, 4.88) | 1.67(0.55, 5.4) | 0.94(0.28, 3.28) | 0.82(0.21, 3.39) | 0.02(0, 0.1) | 0.54(0.15, 1.85) | 1.43(0.36, 5.92) | 0.85(0.29, 2.55) | 1.21(0.41, 3.73) | 1.22(0.36, 4.39) | 0.45(0.01, 5.18) | 1.77(0.63, 5.27) | 0.92(0.32, 2.76) | 1.64(0.47, 6.09) | 2.42(0.84, 7.47) | 1.09(0.2, 6.22) | 1.47(0.14, 18.27) | 0.96(0.32, 2.97) | 0.92(0.24, 3.62) | 1.64(0.47, 6.09) | 1.36(0.37, 5.39) | 1.63(0.45, 6.19) | 2.14(1.19, 5.87) | 0.84(0.49, 2.76) | |
| liraglutide | 1.87(0.32, 11.59) | 0.8(0.3, 2.14) | 0.77(0.21, 2.77) | 0.66(0.2, 2.1) | NA | 1.11(0.41, 3.08) | 0.63(0.21, 1.91) | 0.55(0.14, 2.11) | 0.02(0, 0.07) | 0.36(0.1, 1.12) | 0.95(0.25, 3.61) | 0.56(0.21, 1.48) | 0.81(0.3, 2.22) | 0.81(0.26, 2.65) | 0.3(0.01, 3.22) | 1.18(0.45, 3.15) | 0.61(0.23, 1.64) | 1.09(0.34, 3.67) | 1.6(0.65, 4.23) | 0.73(0.14, 3.85) | 0.97(0.09, 11.85) | 0.64(0.23, 1.79) | 0.61(0.17, 2.21) | 1.09(0.34, 3.67) | 0.9(0.26, 3.22) | 1.08(0.36, 3.33) | 1.40(1.02, 3.02) | 0.74(0.34, 1.66) | |
| semaglutide | 1.69(0.29, 10) | 0.73(0.26, 2.02) | 0.7(0.19, 2.46) | 0.6(0.19, 1.83) | 0.91(0.32, 2.4) | NA | 0.57(0.18, 1.68) | 0.5(0.13, 1.83) | 0.01(0, 0.06) | 0.32(0.09, 1) | 0.86(0.23, 3.17) | 0.51(0.2, 1.24) | 0.73(0.27, 1.95) | 0.74(0.23, 2.28) | 0.27(0.01, 2.91) | 1.07(0.42, 2.64) | 0.55(0.22, 1.4) | 0.98(0.31, 3.14) | 1.45(0.57, 3.75) | 0.66(0.12, 3.37) | 0.88(0.08, 10.44) | 0.57(0.21, 1.54) | 0.55(0.16, 1.94) | 0.98(0.31, 3.14) | 0.81(0.24, 2.84) | 0.98(0.3, 3.19) | 1.29(1.02, 3.23) | 0.67(0.32, 1.4) | |
| vildagliptin | 2.98(0.5, 18.92) | 1.28(0.44, 3.79) | 1.23(0.33, 4.7) | 1.07(0.3, 3.56) | 1.6(0.52, 4.85) | 1.77(0.59, 5.45) | NA | 0.87(0.22, 3.54) | 0.03(0, 0.11) | 0.57(0.16, 1.92) | 1.52(0.39, 6.1) | 0.9(0.32, 2.51) | 1.29(0.44, 3.78) | 1.29(0.39, 4.52) | 0.47(0.01, 5.28) | 1.89(0.66, 5.36) | 0.98(0.35, 2.81) | 1.73(0.51, 6.09) | 2.57(0.94, 7.21) | 1.16(0.22, 6.05) | 1.54(0.15, 19.16) | 1.01(0.37, 2.83) | 0.97(0.26, 3.65) | 1.73(0.51, 6.09) | 1.44(0.4, 5.32) | 1.73(0.57, 5.5) | 2.19(1.12, 6.74) | 1.19(0.51, 2.84) | |
| omarigliptin | 3.39(0.5, 24.34) | 1.46(0.38, 5.62) | 1.4(0.3, 6.71) | 1.21(0.3, 4.78) | 1.84(0.48, 6.87) | 2.01(0.55, 7.52) | 1.14(0.28, 4.51) | NA | 0.04(0, 0.12) | 0.65(0.15, 2.58) | 1.74(0.37, 7.99) | 1.03(0.28, 3.61) | 1.47(0.41, 5.3) | 1.48(0.36, 6.1) | 0.54(0.02, 6.58) | 2.15(0.62, 7.46) | 1.12(0.32, 3.89) | 1.98(0.47, 8.44) | 2.92(0.87, 10.26) | 1.33(0.21, 8.35) | 1.77(0.15, 24.22) | 1.15(0.33, 4.16) | 1.11(0.25, 4.96) | 1.98(0.47, 8.44) | 1.65(0.38, 7.3) | 1.96(0.47, 8.54) | 2.51(1.2, 7.67) | 1.35(0.46, 4.01) | |
| trelagliptin | 4.03(2.84, 16.74) | 5.52(2.21, 13.04) | 5.84(1.38, 12.90) | 4.85(1.59, 12.43) | 3.22(1.16, 13.44) | 5.41(1.98, 17.33) | 4.79(1.46, 12.36) | 3.69(1.12, 17.78) | NA | 2.17(1.51, 6.22) | 4.21(2.27, 13.45) | 3.71(1.67, 11.98) | 5.13(2.68, 23.15) | 5.62(2.33, 13.05) | 3.81(1.21, 11.92) | 4.50(1.53, 14.02) | 4.78(1.35, 12.15) | 5.57(2.71, 24.19) | 9.47(5.36, 33.80) | 3.46(1.74, 13.74) | 6.12(3.9, 28.33) | 4.81(1.87, 15.26) | 4.80(1.33, 12.31) | 3.93(1.71, 14.19) | 4.53(2.15, 13.11) | 6.14(2.29, 23.88) | 7.69(2.45, 23.83) | 3.44(1.41, 12.62) | |
| saxagliptin | 5.24(0.89, 35.52) | 2.25(0.7, 7.77) | 2.17(0.54, 9.2) | 1.87(0.54, 6.5) | 2.78(0.88, 9.51) | 3.1(1, 10.66) | 1.76(0.52, 6.27) | 1.54(0.39, 6.67) | 0.06(0, 0.19) | NA | 2.66(0.71, 10.8) | 1.58(0.55, 4.86) | 2.26(0.77, 7.15) | 2.27(0.68, 8.29) | 0.83(0.02, 9.72) | 3.31(1.16, 10.23) | 1.72(0.59, 5.39) | 3.05(0.99, 10.37) | 4.51(1.63, 14) | 2.03(0.37, 12.14) | 2.72(0.25, 36.33) | 1.77(0.6, 5.89) | 1.71(0.46, 6.88) | 3.05(0.99, 10.37) | 2.53(0.69, 10.18) | 3.03(0.85, 12.01) | 2.41(1.22, 7.38) | 2.08(0.87, 5.53) | |
| alogliptin | 1.95(0.29, 14.05) | 0.84(0.22, 3.27) | 0.81(0.17, 3.8) | 0.7(0.17, 2.75) | 1.04(0.27, 3.96) | 1.17(0.32, 4.34) | 0.66(0.16, 2.57) | 0.57(0.13, 2.69) | 0.02(0, 0.07) | 0.38(0.09, 1.41) | NA | 0.59(0.18, 1.94) | 0.85(0.23, 2.98) | 0.85(0.22, 3.41) | 0.31(0.01, 3.9) | 1.24(0.35, 4.28) | 0.64(0.18, 2.26) | 1.14(0.39, 3.48) | 1.69(0.49, 5.93) | 0.76(0.13, 4.73) | 1.02(0.09, 13.98) | 0.66(0.19, 2.43) | 0.64(0.15, 2.79) | 1.14(0.39, 3.48) | 0.95(0.23, 4.08) | 1.13(0.27, 5.05) | 1.41(1.12, 6.76) | 0.78(0.26, 2.36) | |
| sitagliptin | 3.32(0.62, 19.84) | 1.43(0.56, 3.68) | 1.37(0.39, 4.78) | 1.18(0.39, 3.4) | 1.78(0.67, 4.65) | 1.96(0.81, 4.92) | 1.11(0.4, 3.09) | 0.97(0.28, 3.52) | 0.03(0, 0.12) | 0.63(0.21, 1.81) | 1.69(0.51, 5.57) | NA | 1.43(0.57, 3.64) | 1.44(0.5, 4.23) | 0.53(0.02, 5.57) | 2.1(0.9, 4.86) | 1.09(0.45, 2.68) | 1.93(0.75, 5.19) | 2.85(1.21, 7.01) | 1.29(0.26, 6.35) | 1.72(0.18, 19.96) | 1.13(0.46, 2.81) | 1.08(0.35, 3.43) | 1.93(0.75, 5.19) | 1.6(0.53, 4.91) | 1.92(0.64, 5.98) | 2.45(1.38, 8.69) | 1.32(0.69, 2.59) | |
| linagliptin | 2.31(0.42, 13.67) | 1(0.36, 2.79) | 0.95(0.28, 3.34) | 0.82(0.27, 2.45) | 1.24(0.45, 3.38) | 1.37(0.51, 3.75) | 0.78(0.26, 2.29) | 0.68(0.19, 2.44) | 0.02(0, 0.08) | 0.44(0.14, 1.3) | 1.18(0.34, 4.27) | 0.7(0.28, 1.75) | NA | 1.01(0.34, 3.11) | 0.37(0.01, 3.76) | 1.46(0.59, 3.6) | 0.76(0.32, 1.82) | 1.35(0.44, 4.28) | 1.99(0.97, 4.29) | 0.9(0.18, 4.54) | 1.2(0.12, 14.35) | 0.78(0.31, 2.04) | 0.75(0.23, 2.54) | 1.35(0.44, 4.28) | 1.11(0.36, 3.74) | 1.34(0.43, 4.39) | 2.17(1.04, 5.46) | 0.92(0.47, 1.86) | |
| dapagliflozin | 2.3(0.37, 14.98) | 0.99(0.31, 3.16) | 0.95(0.25, 3.49) | 0.82(0.23, 2.76) | 1.23(0.37, 3.95) | 1.35(0.44, 4.28) | 0.77(0.22, 2.56) | 0.67(0.16, 2.75) | 0.01(0, 0.08) | 0.44(0.12, 1.46) | 1.17(0.29, 4.47) | 0.69(0.24, 1.99) | 0.99(0.32, 2.95) | NA | 0.36(0.01, 4.13) | 1.45(0.49, 4.19) | 0.76(0.26, 2.21) | 1.34(0.42, 4.27) | 1.98(0.71, 5.66) | 0.89(0.16, 4.96) | 1.19(0.11, 14.95) | 0.78(0.26, 2.37) | 0.75(0.2, 2.87) | 1.34(0.42, 4.27) | 1.11(0.3, 4.2) | 1.33(0.37, 4.76) | 1.74(1.34, 6.02) | 0.91(0.38, 2.24) | |
| ertugliflozin | 6.5(0.4, 25.54) | 2.71(0.24, 8.92) | 2.64(0.21, 9.36) | 2.25(0.19, 7.63) | 3.35(0.31, 19.28) | 3.73(0.34, 12.34) | 2.12(0.19, 7.81) | 1.87(0.15, 6.79) | 0.1(0, 0.31) | 1.2(0.1, 41.23) | 3.24(0.26, 13.25) | 1.89(0.18, 6.7) | 2.7(0.27, 8.38) | 2.75(0.24, 9.77) | NA | 3.97(0.38, 12.32) | 2.06(0.2, 7.34) | 3.68(0.32, 12.27) | 5.39(0.56, 16.04) | 2.5(0.17, 9.21) | 3.49(0.14, 20.33) | 2.14(0.2, 7.07) | 2.08(0.17, 7.59) | 3.68(0.32, 12.27) | 3.06(0.26, 16.51) | 3.66(0.31, 15.59) | 7.50(2.03, 21.85) | 2.49(0.26, 7.55) | |
| canagliflozin | 1.58(0.29, 9.19) | 0.68(0.26, 1.84) | 0.65(0.19, 2.3) | 0.56(0.19, 1.6) | 0.85(0.32, 2.26) | 0.94(0.38, 2.37) | 0.53(0.19, 1.52) | 0.47(0.13, 1.61) | 0.01(0, 0.05) | 0.3(0.1, 0.86) | 0.81(0.23, 2.82) | 0.48(0.21, 1.11) | 0.68(0.28, 1.69) | 0.69(0.24, 2.03) | 0.25(0.01, 2.64) | NA | 0.52(0.22, 1.23) | 0.92(0.31, 2.83) | 1.36(0.6, 3.24) | 0.61(0.12, 3.05) | 0.82(0.08, 9.83) | 0.54(0.22, 1.36) | 0.52(0.16, 1.72) | 0.92(0.31, 2.83) | 0.76(0.24, 2.52) | 0.91(0.3, 2.93) | 1.21(1.02, 3.90) | 0.63(0.34, 1.19) | |
| empagliflozin | 3.04(0.56, 17.75) | 1.31(0.48, 3.57) | 1.26(0.36, 4.36) | 1.09(0.36, 3.09) | 1.62(0.6, 4.32) | 1.8(0.71, 4.62) | 1.02(0.36, 2.9) | 0.89(0.26, 3.1) | 0.03(0, 0.11) | 0.58(0.19, 1.69) | 1.55(0.44, 5.42) | 0.92(0.37, 2.23) | 1.32(0.55, 3.16) | 1.32(0.45, 3.91) | 0.48(0.01, 4.97) | 1.92(0.81, 4.59) | NA | 1.76(0.59, 5.53) | 2.61(1.17, 6.1) | 1.19(0.24, 5.83) | 1.58(0.16, 18.62) | 1.03(0.42, 2.56) | 0.99(0.31, 3.24) | 1.76(0.59, 5.53) | 1.46(0.48, 4.68) | 1.76(0.58, 5.62) | 2.29(1.57, 7.70) | 1.21(0.65, 2.29) | |
| glipizide | 1.71(0.28, 11.39) | 0.74(0.22, 2.42) | 0.71(0.17, 2.89) | 0.61(0.16, 2.13) | 0.92(0.27, 2.98) | 1.02(0.32, 3.22) | 0.58(0.16, 1.94) | 0.5(0.12, 2.12) | 0.02(0, 0.06) | 0.33(0.1, 1.01) | 0.88(0.29, 2.57) | 0.52(0.19, 1.33) | 0.74(0.23, 2.29) | 0.74(0.23, 2.39) | 0.27(0.01, 3.14) | 1.09(0.35, 3.21) | 0.57(0.18, 1.69) | NA | 1.48(0.49, 4.47) | 0.66(0.12, 3.72) | 0.89(0.08, 11.05) | 0.58(0.18, 1.81) | 0.56(0.16, 2.01) | 2.01(0.56, 7.41) | 0.83(0.23, 3.01) | 0.99(0.27, 3.76) | 1.27(1.06, 4.23) | 0.69(0.27, 1.74) | |
| glimepiride | 1.16(0.21, 6.69) | 0.5(0.18, 1.28) | 0.48(0.15, 1.47) | 0.41(0.13, 1.18) | 0.62(0.24, 1.54) | 0.69(0.27, 1.77) | 0.39(0.14, 1.06) | 0.34(0.1, 1.15) | 0.01(0, 0.04) | 0.22(0.07, 0.61) | 0.59(0.17, 2.05) | 0.35(0.14, 0.83) | 0.5(0.23, 1.03) | 0.51(0.18, 1.41) | 0.19(0.01, 1.79) | 0.73(0.31, 1.66) | 0.38(0.16, 0.85) | 0.68(0.22, 2.05) | NA | 0.45(0.09, 2.13) | 0.6(0.06, 7) | 0.39(0.16, 0.94) | 0.38(0.12, 1.24) | 0.68(0.22, 2.05) | 0.56(0.18, 1.8) | 0.67(0.23, 2.02) | 1.18(1.09, 3.05) | 0.46(0.24, 0.88) | |
| glibenclamide | 2.58(0.3, 23.36) | 1.11(0.21, 5.87) | 1.06(0.17, 6.43) | 0.92(0.16, 5.06) | 1.38(0.26, 7.21) | 1.52(0.3, 8.02) | 0.86(0.17, 4.52) | 0.75(0.12, 4.75) | 0.03(0, 0.09) | 0.49(0.08, 2.67) | 1.32(0.21, 7.89) | 0.78(0.16, 3.78) | 1.11(0.22, 5.51) | 1.13(0.2, 6.29) | 0.4(0.01, 6) | 1.63(0.33, 8.02) | 0.84(0.17, 4.19) | 1.5(0.27, 8.51) | 2.23(0.47, 10.82) | NA | 1.33(0.11, 18.97) | 0.87(0.23, 3.23) | 0.84(0.15, 4.81) | 1.5(0.27, 8.51) | 1.24(0.22, 7.12) | 1.49(0.26, 8.52) | 7.09(2.86, 22.31) | 1.02(0.23, 4.52) | |
| gliclazide | 1.94(0.11, 30.75) | 0.83(0.07, 8.82) | 0.8(0.06, 9.21) | 0.68(0.05, 7.3) | 1.03(0.08, 10.68) | 1.14(0.1, 11.83) | 0.65(0.05, 6.75) | 0.56(0.04, 6.59) | 0.02(0, 0.07) | 0.37(0.03, 3.99) | 0.98(0.07, 11.58) | 0.58(0.05, 5.62) | 0.83(0.07, 8.12) | 0.84(0.07, 9.2) | 0.29(0, 7.34) | 1.22(0.1, 12.1) | 0.63(0.05, 6.22) | 1.12(0.09, 11.94) | 1.67(0.14, 16.4) | 0.75(0.05, 9.32) | NA | 0.66(0.06, 5.74) | 0.63(0.06, 5.85) | 1.12(0.09, 11.94) | 0.94(0.08, 9.03) | 1.12(0.09, 12.66) | 1.77(1.21, 5.58) | 0.77(0.07, 7.13) | |
| pioglitazone | 2.96(0.53, 17.22) | 1.27(0.45, 3.54) | 1.22(0.34, 4.37) | 1.05(0.34, 3.09) | 1.57(0.56, 4.37) | 1.74(0.65, 4.75) | 0.99(0.35, 2.71) | 0.87(0.24, 3.07) | 0.03(0, 0.1) | 0.56(0.17, 1.66) | 1.5(0.41, 5.37) | 0.89(0.36, 2.16) | 1.28(0.49, 3.24) | 1.28(0.42, 3.89) | 0.47(0.01, 4.89) | 1.86(0.74, 4.57) | 0.97(0.39, 2.37) | 1.72(0.55, 5.41) | 2.53(1.06, 6.16) | 1.14(0.31, 4.28) | 1.52(0.17, 16.01) | NA | 0.96(0.3, 3.09) | 1.72(0.55, 5.41) | 1.42(0.46, 4.5) | 1.71(0.54, 5.5) | 2.25(1.19, 7.08) | 1.17(0.59, 2.35) | |
| rosiglitazone | 3.08(0.46, 21.26) | 1.32(0.38, 4.52) | 1.26(0.28, 5.5) | 1.09(0.28, 4.11) | 1.64(0.46, 5.8) | 1.82(0.52, 6.3) | 1.03(0.27, 3.79) | 0.9(0.2, 3.94) | 0.03(0, 0.11) | 0.58(0.15, 2.16) | 1.56(0.36, 6.55) | 0.92(0.29, 2.87) | 1.33(0.39, 4.29) | 1.34(0.35, 5.06) | 0.48(0.01, 5.76) | 1.94(0.58, 6.26) | 1.01(0.31, 3.17) | 1.78(0.5, 6.44) | 2.65(0.8, 8.61) | 1.19(0.21, 6.83) | 1.58(0.17, 17.62) | 1.04(0.32, 3.35) | NA | 1.78(0.5, 6.44) | 1.48(0.75, 2.91) | 1.77(0.45, 7.16) | 2.39(1.82, 7.77) | 1.22(0.43, 3.4) | |
| nateglinide | 3.45(0.57, 22.13) | 1.49(0.44, 4.93) | 1.43(0.34, 5.95) | 1.24(0.34, 4.12) | 1.85(0.55, 5.93) | 2.04(0.64, 6.56) | 1.16(0.33, 3.98) | 1.01(0.25, 4.16) | 0.03(0, 0.12) | 0.66(0.17, 2.25) | 1.77(0.42, 7.13) | 1.04(0.34, 3.09) | 1.49(0.47, 4.54) | 1.51(0.42, 5.27) | 0.54(0.02, 6.23) | 2.19(0.72, 6.47) | 1.14(0.38, 3.34) | 2.01(0.56, 7.41) | 2.96(0.99, 9.14) | 1.34(0.24, 7.51) | 1.8(0.16, 22.89) | 1.17(0.37, 3.63) | 1.12(0.29, 4.39) | NA | 1.66(0.43, 6.45) | 2(0.53, 7.64) | 2.59(1.24, 8.71) | 1.38(0.56, 3.31) | |
| metformin | 2.07(0.31, 14.23) | 0.89(0.26, 2.98) | 0.86(0.19, 3.68) | 0.74(0.19, 2.74) | 1.11(0.31, 3.79) | 1.23(0.35, 4.14) | 0.69(0.19, 2.5) | 0.61(0.14, 2.64) | 0.02(0, 0.07) | 0.4(0.1, 1.44) | 1.06(0.25, 4.37) | 0.62(0.2, 1.89) | 0.9(0.27, 2.81) | 0.9(0.24, 3.34) | 0.33(0.01, 3.84) | 1.31(0.4, 4.15) | 0.68(0.21, 2.07) | 1.2(0.33, 4.37) | 1.79(0.56, 5.64) | 0.81(0.14, 4.57) | 1.07(0.11, 12.31) | 0.7(0.22, 2.18) | 0.67(0.34, 1.34) | 1.2(0.33, 4.37) | NA | 1.2(0.31, 4.7) | 1.61(1.16, 5.15) | 0.83(0.29, 2.26) | |
| insulin | 1.73(0.27, 11.6) | 0.74(0.29, 1.81) | 0.71(0.21, 2.29) | 0.61(0.16, 2.21) | 0.93(0.3, 2.75) | 1.02(0.31, 3.31) | 0.58(0.18, 1.76) | 0.51(0.12, 2.13) | 0.02(0, 0.06) | 0.33(0.08, 1.18) | 0.88(0.2, 3.69) | 0.52(0.17, 1.55) | 0.75(0.23, 2.33) | 0.75(0.21, 2.67) | 0.27(0.01, 3.22) | 1.1(0.34, 3.36) | 0.57(0.18, 1.73) | 1.01(0.27, 3.77) | 1.49(0.5, 4.42) | 0.67(0.12, 3.8) | 0.89(0.08, 11.46) | 0.59(0.18, 1.85) | 0.57(0.14, 2.2) | 1.01(0.27, 3.77) | 0.83(0.21, 3.24) | NA | 1.33(1.18, 4.38) | 0.69(0.25, 1.81) | |
| voglibose | 0.15(0, 0.56) | 0.08(0, 0.23) | 0.06(0, 0.22) | 0.06(0, 0.19) | 0.08(0, 0.23) | 0.1(0, 0.31) | 0.06(0, 0.17) | 0.04(0, 0.15) | 0.01(0, 0.04) | 0.03(0, 0.1) | 0.08(0, 0.28) | 0.05(0, 0.16) | 0.08(0, 0.23) | 0.08(0, 0.23) | 0.03(0, 0.09) | 0.11(0, 0.32) | 0.06(0, 0.17) | 0.1(0, 0.31) | 0.13(0, 0.44) | 0.06(0, 0.21) | 0.1(0, 0.31) | 0.06(0, 0.18) | 0.05(0, 0.17) | 0.1(0, 0.31) | 0.08(0, 0.25) | 0.1(0, 0.31) | NA | 0.06(0, 0.2) | |
| placebo | 2.51(1.52, 12.84) | 1.08(0.48, 2.39) | 1.03(0.34, 3.18) | 1.12(0.36, 2.05) | 1.35(0.6, 2.92) | 1.49(0.71, 3.14) | 0.84(0.35, 1.96) | 0.74(0.25, 2.15) | 0.03(0, 0.09) | 0.48(0.18, 1.15) | 1.28(0.42, 3.79) | 0.76(0.39, 1.45) | 1.09(0.54, 2.15) | 1.09(0.45, 2.65) | 0.4(0.01, 3.88) | 1.59(0.84, 2.91) | 0.83(0.44, 1.53) | 1.46(0.57, 3.77) | 2.16(1.13, 4.23) | 0.98(0.22, 4.27) | 1.3(0.14, 14.35) | 0.85(0.43, 1.69) | 0.82(0.29, 2.31) | 1.46(0.57, 3.77) | 1.21(0.44, 3.41) | 1.45(0.55, 3.96) | 7.19(2.89, 26.25) | NA | |
| Comparisons should be read from left to right. The estimate is located at the intersection of the treatments in the column heads and the treatments in the row heads. An RR value >1 favors the column-defining treatment. An RR value <1 favors the row-defining treatment; NA: not applicable. | | | | | | | | | | | | | | | | | | | | | | | | | | | | |  |
